# SCO6564, a novel 3-ketoacyl acyl carrier protein synthase III, contributes in fatty acid synthesis in *Streptomyces coelicolor*

**DOI:** 10.1371/journal.pone.0318258

**Published:** 2025-02-06

**Authors:** Jian-Rong Ma, Jia-Ying Lin, Yuan-Yin Zhang, Yun Chen, Wen-Bing Zhang, Xian-Pu Ni, Yong-Hong Yu

**Affiliations:** 1 Guangdong Food and Drug Vocational College, Guangzhou, Guangdong, China; 2 College of Life Science and Biopharmaceutics, Shenyang Pharmaceutical University, Shenyang, Liaoning, China; 3 College of Life Sciences, South China Agricultural University, Guangzhou, Guangdong, China; Lahore University of Management Sciences, PAKISTAN

## Abstract

The genus *Streptomyces* comprises gram-positive bacteria that produce large numbers of secondary metabolites, which have promising commercial applications and deserve extensive study. Most bacteria synthesize fatty acids using a type II fatty acid synthase, with each step catalyzed by a discrete protein. Fatty acid synthesis has been intensively studied in the model strain *Streptomyces coelicolor*, in which 3-ketoacyl-acyl carrier protein synthase III (KAS III, FabH) is essential for growth and fatty acid biosynthesis. In this study, the FabH homolog SCO6564 (named FabH2) was identified in the *S*. *coelicolor* genome by BLAST analysis. The expression of *fabH2* restored the growth of *Ralstonia solanacearum fabH* mutant and made the mutant produce small amounts of branched-chain fatty acids. FabH2 could condense various substrates, including straight-chain and branched-chain acyl-CoAs, with malonyl-acyl carrier protein to initiate fatty acid synthesis in *in vitro* assays. The *fabH2* deletion did not cause significant changes in the growth or fatty acid composition of *S*. *coelicolor*, indicating that *fabH2* is nonessential for growth or fatty acid synthesis. However, *fabH2* overexpression reduced the blue-pigmented actinorhodin production. Phylogenetic analysis of KAS III from different bacteria revealed that FabH2 belongs to a novel group of FabH-type, which is ubiquitous in *Streptomyces* spp.

## Introduction

Streptomycetes, a gram-positive filamentous bacteria with a high G + C content in chromosomal DNA, produces many types of secondary metabolites that are widely used in human health, including >75% of the antibiotics used as medicine, and many compounds containing antiviral, antiparasitic, anticancer, and immunosuppressive activities [1–3]. Although the secondary metabolites are nonessential for bacterial growth, they are believed to play significant roles in their natural habitats. Streptomycetes strains can be found in various terrestrial and aquatic environments, including extreme habitats [4, 5]. *Streptomyces coelicolor* is regarded as the model strain for extensive study [6], because it produces well-characterized antibiotics, including the blue-pigmented polyketide actinorhodin (ACT) and the red-pigmented undecylprodigiosin (RED). ACT has served as an outstanding example for genetic and biochemical investigations of polyketide metabolism [7], whereas RED is a representative product synthesized using fatty acids as a building block, similar to the biosynthesis of many natural products in Streptomycetes [8].

Bacteria, such as Streptomycetes, utilize type II fatty acid synthase (FAS II) to synthesize fatty acids, involving a series of enzymes encoded by discrete genes [9]. By contrast, mammals and fungi use multifunctional fatty acid synthase I to catalyze fatty acid synthesis [10]. FAS II not only provides substrates for the biosynthesis of phospholipids, lipoproteins, and lipopolysaccharides, but also supplies intermediates for the synthesis of many important bioactive products, such as lipoic acid and biotin [11, 12]. Moreover, FAS II is involved in the production of different quorum sensing signals, such as *N*-acyl homoserine lactones [13] and diffusible signal factors [14, 15]. Thus, FAS II is believed to be a promising target for antibacterial discovery [16].

Bacterial pathway of fatty acids synthesis can be divided into two stages as follows: initiation and elongation. The initiation stage *de novo* produces 3-ketoacyl ACP by condensation of an acetyl-CoA and malonyl-ACP, which is elongated into long-chain fatty acids with condensation, reduction, dehydration, and reduction cycles [17]. The final products of FAS II will be transferred to glycerol-3-P by PlsX/Y or PlsB/C, resulting in the formation of membrane phospholipid. Bacterial membrane homeostasis is greatly determined by the structures of the fatty acids chains, in which straight-chain saturated fatty acids (SCFAs) are linear and pack together efficiently to produce a bilayer that has a high phase transition and low permeability properties. While unsaturated fatty acids (UFAs) and branched-chain fatty acids (BCFAs) incorporation results in lower transition temperatures and higher permeability [18, 19].

The initiation mechanisms of fatty acid synthesis vary in different bacteria. FabH, a 3-ketoacyl ACP synthetase III (KAS III), was first identified as the key enzyme involved in fatty acid synthesis initiation in the model organism *Escherichia coli* [20]. FabH homologs were found in the fatty acid synthetic loci in many bacterial genomes, indicating that the initiation mechanism catalyzed by FabH-type KAS III is relatively conserved across bacteria. However, FabHs from different bacteria show different substrate selectivity. FabH from *E*. *coli* and *Ralstonia solanacearum* only accept acetyl-CoA as substrate, thus synthesizing SCFAs [21, 22]. By contrast, FabH from gram-positive bacteria, such as *Bacillus subtilis* [23, 24] and *S*. *coelicolor* [8, 25], have a strong preference for branched-chain acyl-CoAs as the primers, producing BCFAs in these bacteria. Our group reported that FabHs from the gram-negative phytopathogen *Xanthomonas campestris* pv. *campestris* (*Xcc*) [26] and *X*. *oryzae* pv. *oryzae* (*Xoo*) [14] can utilize branched-chain primers to synthesize BCFAs. The opportunistic pathogen *Pseudomonas aeruginosa* encodes no FabH homolog, but utilizes FabY-type and PA3286-type KAS III enzymes to initiate fatty acid synthesis. FabY has similar activity to *E*. *coli* FabH, but exhibits different structural features [27], whereas PA3286 has a broader substrate specificity, because it can utilize octanoyl-CoA to initiate fatty acid synthesis [28]. Although Rsp0194 functions like PA3286-type KAS III, *R*. *solanacearum* can alternatively utilize medium-chain acyl-CoAs as primers to initiate fatty acid synthesis [22]. Recently, our group reported that FabH1 of *P*. *syringae* pv. *syringae* B728a is an atypical KAS III that functions in providing a critical fatty acid precursor, butyryl-ACP, for *N*-acyl homoserine lactone synthesis [29]. Guo *et al*. demonstrated that *P*. *putida* can use the long-chain KAS I (FabB) to catalyze the initiation reaction in FAS II [30]. Another type of initiation reaction, the malonyl-ACP decarboxylase (MAD) pathway, was identified in *E*. *coli* and *P*. *putida*, in which MAD can bypass or replace the KAS III pathway [31, 32]. In the MAD pathway, malonyl-ACP is converted to acetyl-ACP by MAD, which is further condensed with malonyl-ACP by the long-chain KAS I (FabB) or KAS II (FabF) to form the initial 3-ketobutyryl-ACP substrate used in the subsequent elongation reactions [33]. The MAD pathway complements, bypasses, or replaces the KAS III pathway in some bacteria. In summary, the initiation of fatty acid synthesis shows a large diversity, and one bacterial strain can use different pathways in FAS II.

*S*. *coelicolor* FabH (hereafter renamed *Sco* FabH1) was first identified by Hopwood *et al*. [25]. Reynolds *et al*. confirmed its greater catalytic efficiency to branched-chain primers [8]. Moreover, our group estabolished *Sco* FabH1’s activity in the initiation of fatty acid synthesis by genetic complementation and *in vitro* analysis [34]. Because *Sco fabH1* is essential for growth, Li *et al*. generated the *fabH1* deletion mutant by plasmid-based expression of *E*. *coli fabH*. This strain grew much slower, but produced ~14% of the BCFAs [35]. Our team studied *Xcc fabH* using a similar strategy, but the *Xcc fabH* deletion mutant, in which *E*. *coli fabH* was expressed from plasmid pSRK-Gm, produced only trace amounts (<1%) of BCFAs [26]. *E*. *coli* FabH can only synthesize SCFAs, then how BCFAs were synthesized in the *S*. *coelicolor fabH1* deletion mutant complemented with *E*. *coli fabH* [35]? Thus, we hypothesize that there is another pathway to initiate BCFAs synthesis in *S*. *coelicolor*.

Here, we studied *SCO6564* in the genome of *S*. *coelicolor* by employing different molecular biological techniques, including bioinformatic analysis, genetic complementation, biochemical analyses, and gene deletion. The results demonstrated that *SCO6564* encodes an active KAS III, named *Sco* FabH2, which contains the conserved triplet Cys–His–Asn catalytic site. *Sco* FabH2 has catalytic activities toward various substrates, but plays a minor role in fatty acid biosynthesis in *S*. *coelicolor*. *Sco* FabH2 represents a novel FabH-type KAS III, which is mainly present in *Streptomyces* spp.

## Materials and methods

### Strains and culture conditions

The strains and plasmids used in this study are listed in S1 Table in S1 File. The *E*. *coli* strains were cultured at 37°C in LB Miller medium (10 g of tryptone, 5 g of yeast extract, and 10 g of NaCl per liter, pH 7.0). *R*. *solanacearum* strains were grown at 30°C in BG medium (10 g of bacto peptone, 1 g of yeast extract, 1 g of casamino acids, 5 g of glucose per liter, pH 7.0). The *S*. *coelicolor* strains were cultured at 28°C in YBP (for ACT and RED testing, 4 g of tryptone, 2 g of yeast extract, 2 g of beef extract, 10 g of glucose, 15 g of NaCl, and 1 g of MgSO_4_•7H_2_O per liter, pH 7.2) [36] or Gauze’s synthetic medium no. 1 (for sporulation, 20 g of soluble starch, 0.1 g of beef extract, 0.1 g of KNO_3_, 0.05 g of K_2_HPO_4_•3H_2_O, 0.05 g of NaCl, 0.05 g of MgSO_4_•7H_2_O, 0.001 g of FeSO_4_•7H_2_O per liter [pH 7.0–7.2]) [37]. When required, the following antibiotics were added (per mL): 100 μg of sodium ampicillin, 30 μg of kanamycin sulfate, 30 μg of chloramphenicol, 50 μg of apramycin, 100 μg of erythromycin.

### Gene cloning and construction of expression plasmids

*coelicolor fabH1*, *fabH2*, *fabH3*, and *fabH4* were amplified from the genomic DNA of the wild-type (WT) strain using UP-DN primer pairs (listed in S2 Table in S1 File). The PCR fragments were purified, digested with *Nde* I and *Hin*d III, and ligated into the pSRK-Km [38] expression vector to obtain the pMJR-1–pMJR-4 plasmid constructs, respectively. *Sco fabH2* was inserted into the *Nde* I and *Hin*d III sites of pET-28(b), using a similar strategy to obtain pMJR-5. *Sco fabH2* was further amplified with the *Sco fabH2*-P1 and *Sco fabH2*-P2 primer pair and cloned into pJY813 between the *Nde* I and *Bgl* II sites to construct plasmid pMJR-9. All the constructs were confirmed by sequencing by Sangon Biotech. Co, Ltd.

### Complementation of the *R*. *solanacearum fabH* deletion strain RsmH

Plasmids pMJR-1–pMJR-4 or empty vector were firstly transferred into *E*. *coli* strain S17-1. Then, the strain was mated with *R*. *solanacearum fabH* deletion strain RsmH [22] on BG plates with octanoic acid (0.1%) for 48 h at 30°C. The cells were suspended in BG medium, and appropriate dilutions were inoculated onto BG plates (with octanoic acid) containing chloramphenicol (to select against the donor strain) plus kanamycin. The transformed strains were inoculated onto BG plates with or without octanoic acid, and growth was determined after 2 d of incubation at 30°C.

### Assay of *Sco* FabH2 activity *in vitro*

pMJR-5 was first transferred into *E*. *coli* BL21(DE3), and *Sco* FabH2 with His_6_-tagged N-terminus was purified with Ni-NTA agarose (Qiagen) using a nickel-ion affinity column (Qiagen). *E*. *coli* FabD, FabH, FabG, FabZ, and FabI and *E*. *coli* holo-ACP proteins were purified as described [29]. To assay the 3-ketoacyl-ACP synthase activity of *Sco* FabH2 *in vitro*, different assay mixtures were prepared, which contained 0.1 M sodium phosphate (pH 7.0), 0.1 μg each of FabD, *Sco* FabH2, FabG, and FabZ, 50 μM NADH, 50 μM NADPH, 1 mM β-mercaptoethanol, 100 μM malonyl-CoA, 50 μM holo-ACP, and 100 μM of substrate (acetyl-CoA, isobutyryl-CoA, isovaleryl-CoA, butyryl-CoA, hexanoyl-CoA, or octanoyl-CoA) in a final volume of 40 μL. The reactions were initiated by adding FabH and followed by incubation for 1 h at 37°C. The reaction products were resolved by conformationally sensitive gel electrophoresis on 20% polyacrylamide gels containing a concentration of urea optimized for separation [39]. The gels were stained with Coomassie Brilliant Blue R-250.

### In-frame chromosomal gene deletion and complementation

The upstream fragment of *Sco fabH2* was amplified using the *Sco fabH2*-1 and *Sco fabH2*-2 primer pair from the chromosomal DNA of *S*. *coelicolor* and ligated into pMD19-T to obtain pMJR-6. The downstream fragment of *Sco fabH2*, amplified with *Sco fabH2*-3 and *Sco fabH2*-4, was inserted and cloned into the *Pst* I and *Eco* RI of pMJR-6 to obtain pMJR-7. The cloning was confirmed by sequencing. The primers used are listed in S2 Table in S1 File. The upstream and downstream fragments of *Sco fabH2* were digested from pMJR-7 with *Eco* RI and *Hin*d III and cloned into the same sites of the vector pKC1139 [40] to construct pMJR-8.

Following conjugation between the derivative of the *E*. *coli* ET12567 strain carrying pMJR-8 with the *S*. *coelicolor* WT strain was conducted on MS medium at 28°C for 20 h. After overlaying with apramycin (50 μg/mL) and nalidixic acid (50 μg/mL), incubation was continued at 28°C for 4–7 d. Single-crossover exconjugants were obtained using apramycin as a selection marker. After three rounds of sporulation in the absence of apramycin, many apramycin-sensitive strains were obtained. Several colonies were chosen randomly for chromosomal DNA extraction. The *Sco fabH2* deletion strain (Δ*fabH2*) was selected out by PCR using the *Sco fabH2*-5 and *Sco fabH2*-6 primer pair and verified by sequencing the PCR products. The pMJR-9 expression plasmid was transferred into the *Sco fabH2* deletion mutant Δ*fabH2* to obtain the complementary strains by conjugation with *E*. *coli* ET12567.

### Analysis of fatty acid profile

The WT and derivative strains of *S*. *coelicolor* were cultured in YBP medium at 28°C for 3 d. The cells were harvested by filtration and washed three times with water. Fatty acid methyl esters were synthesized and extracted as described [41]. Briefly, cellular lipids were saponified by adding 2 mL of a sodium hydroxide and methanol solution at 100°C for 50 min with shaking (800 rpm). The fatty acids were methylated by adding 4 mL of a hydrochloric acid and methanol solution at 80°C for 40 min and immediately cooled to <20°C. The fatty acid methyl esters were obtained by three extractions with 1 mL of petroleum ether. The solvent was removed under a stream of nitrogen, and the residue was analyzed by GC-MS, using *n*-hexane as solvent.

### Examination of ACT production

Different strains of *S*. *coelicolor* spores were inoculated on YBP agar plates, and the plates were incubated at 28°C for 8 d ACT production was quantified as described [42]. Briefly, total ACT was quantified by scraping off all the medium containing mycelia from the plate and mixing with 1 M KOH. The reaction was conducted overnight at 25°C, and then a 1 mL aliquot was used for quantification. Intracellular ACT was quantified using only the mycelia, and extracellular ACT was quantified using only the medium ACT in the sample was quantified by measuring absorbance at 640 nm.

### Statistical analyses

The experimental data were analyzed by analysis of variance using JMP software, version 50 (SAS Institute Inc.). Significant effects of treatment were determined using *F* values (*P* = 005). When *F* value was significant, the means were separated by Fisher’s protected least significant difference at *P* = 005.

## Results

### Bioinformatic analysis with FabH homologs in *S*. *coelicolor*

**Fig 1:**
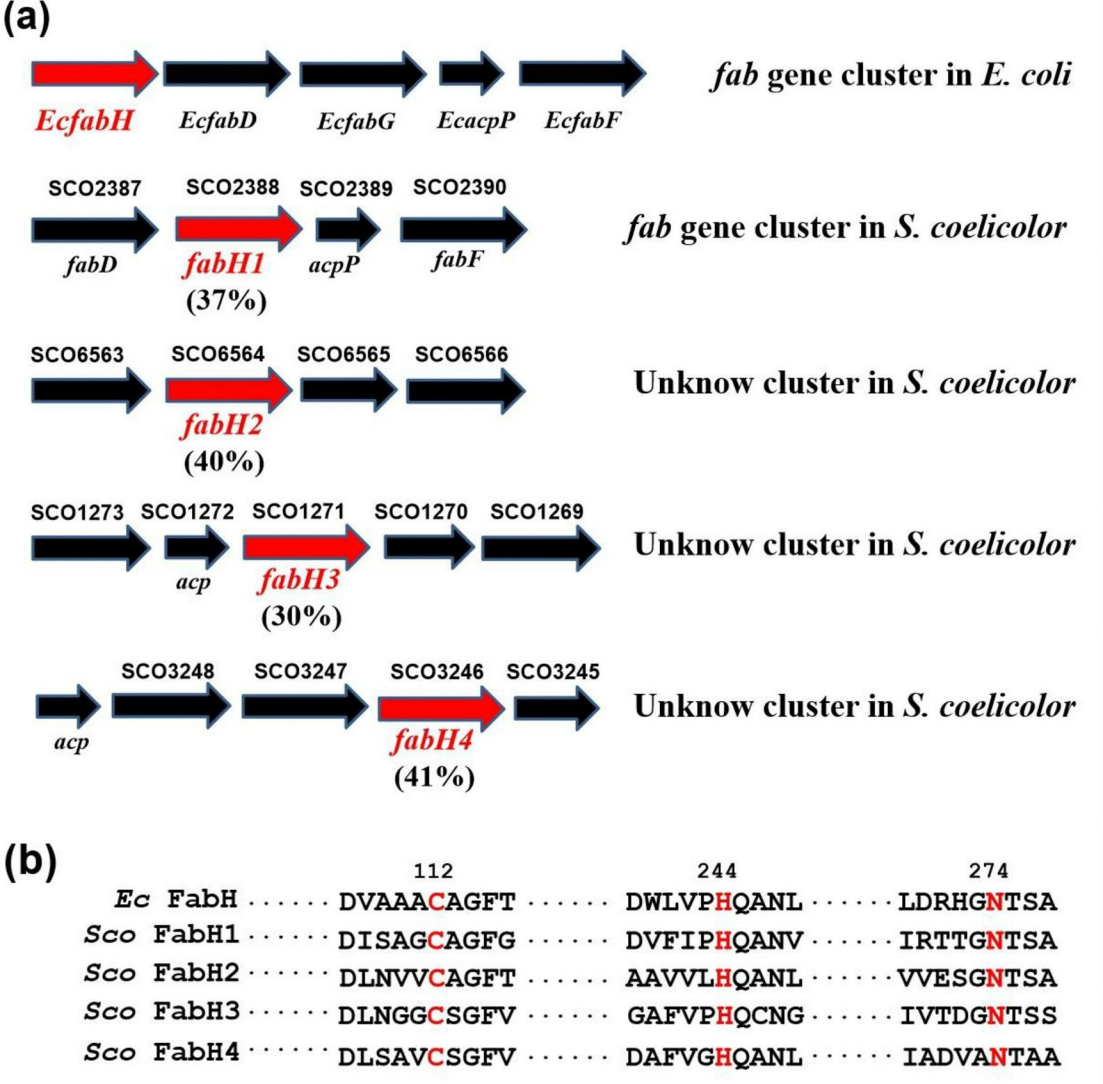
Bioinformatic analysis with FabH homologs in *S*. *coelicolor*. (**a**) Loci of *Sco fabHs*. The *fabH1* locus is similar to the *E*. *coli fabH* locus. (**b**) Structure-based amino acid alignments of *Sco* FabH1–FabH4 with *E*. *coli* FabH (*Ec* FabH). Residues that constitute the Cys (C)–His (H)–Asn (N) catalytic triad are highlighted (Cys_112_, His_244_, and Asn_274_ in *Ec* FabH, respectively).

To investigate alternative initiation pathways of fatty acid synthesis in *S*. *coelicolor*, we performed Blastp analysis of the whole genome with *E*. *coli* FabH (*Ec* FabH) [43] as the query sequence. Four open reading frames were found as FabH homologs, including the reported *Sco fabH1* in the conserved fatty acid biosynthesis (*fab*) gene cluster. The other three, *SCO6564* (*Sco fabH2*), *SCO1271* (*Sco fabH3*), and *SCO3246* (*Sco fabH4*), were among unknown clusters (Fig 1A). The amino acid sequence identities of *Sco* FabH1–FabH4 to *Ec* FabH were 37%, 40%, 30%, and 41%, respectively (Fig 1A). Because *Sco* FabH1 is active and essential for fatty acid synthesis [25, 34], we performed sequence alignment analysis among the four *Sco* FabHs, and found that *Sco* FabH1 shares 41%, 43%, and 38% identity with *Sco* FabH2–FabH4, respectively. The conservative Cys–His–Asn triad motif typical of a KAS III enzyme was found in the four *Sco* FabHs (Fig 1B). The three-dimensional structures of *Sco* FabHs were predicted using SWISS-MODEL (https://swissmodelexpasyorg). All shared structural similarity with *Sco* FabH1 (S1 Fig in S1 File). Overall, the results of bioinformatics analysis strongly suggested that in addition to the reported *Sco* FabH1, the other three *Sco* FabHs may be active in catalyzing the initiation step of fatty acid synthesis in *S*. *coelicolor*.

### *Sco fabH2* could recover the growth of RsmH and produce BCFAs

**Fig 2:**
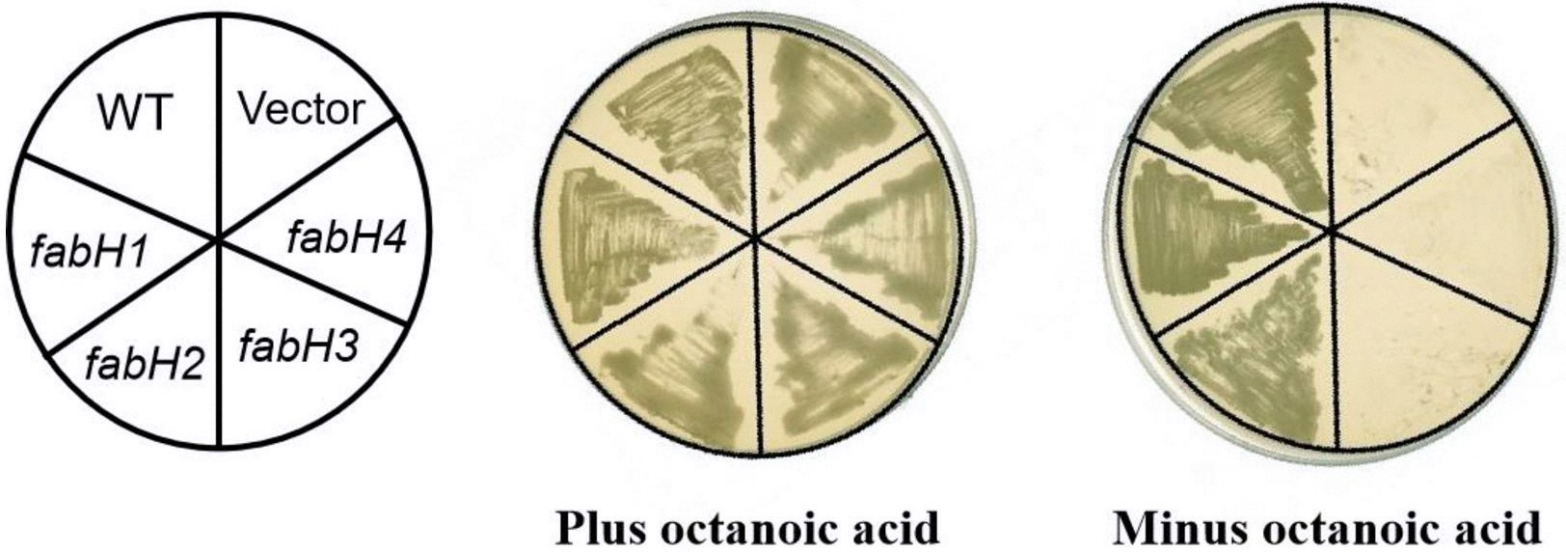
Expression of *Sco fabH1* and *Sco fabH2* restored the growth of *R*. *solanacearum fabH* knockout strain RsmH on BG medium in the absence of octanoic acid.

To study the functions of *Sco* FabHs found in the *S*. *coelicolor* genome with bioinformatics, *Sco fabH1*–*fabH4* were amplified and cloned into the expression vector pSRK-Km under the *E*. *coli lac* promoter to obtain pMJR-1–4. These were further introduced into the *R*. *solanacearum fabH* mutant strain RsmH by conjugation to obtain the transformants RsJR1–RsJR4, respectively. *R*. *solanacearum* RsmH is an octanoic acid auxotrophic mutant, in which the *R*. *solanacearum* genomic *fabH* is deleted in-frame [22]. All the RsmH derivatives grew well on a BG plate containing octanoic acid. Only pMJR-1 (*Sco fabH1*) and pMJR-2 (*Sco fabH2*) introduction could restore the growth of RsmH strain on BG in the absence of octanoic acid, but *Sco fabH3* or *Sco fabH4* expression could not rescue the growth of RsmH under the same condition (Fig 2). These results indicate that both *Sco* FabH1 and *Sco* FabH2, but not *Sco* FabH3 or *Sco* FabH4, were active KAS III to initiate fatty acid synthesis. Because the functions of *Sco* FabH1 have been confirmed in previous reports, we did an in-depth study of FabH2 to determine its functions.

To evaluate the functions of *Sco* FabH2 in fatty acid synthesis, *R*. *solanacearum* and the two RsmH derivatives harboring pMJR-1 (*Sco fabH1*) and pMJR-2 (*Sco fabH2*) were incubated and harvested at 30°C, then their fatty acids were extracted and determined by gas chromatography–mass spectrometry. The *R*. *solanacearum* WT strain GMI1000 does not produce BCFAs. However, the strain synthesized 50% of the UFAs with *n*-C_16:1_ and *n*-C_18:1_, whereas *Sco fabH1* expression caused the RsmH strain to produce ~40% of the BCFAs—*iso*-C_17:0_ and *anteiso*-C_17:0_ were predominant. The content of UFAs in *Sco fabH1*/RsmH was reduced to ~32% (Table 1). By contrast, the RsJR2 (*Sco fabH2)* strain produced similar profiles of fatty acids as the WT strain, producing ~49% of the UFAs. However, minor amounts (~6.4%) of BCFAs, mainly *iso*-C_16:0_ and *iso*-C_17:0_, were detected in RsJR2, indicating that *Sco* FabH2 can catalyze initiation in SCFA and BCFA biosynthesis (Table 1). Because the content of BCFAs produced in RsJR2 was lesser than that in RsJR1, we hypothesize that *Sco* FabH2 is much less active to branched-chain primers than *Sco* FabH1.

**Table 1:** Fatty acid composition of RsmH strains complemented with *Sco fabH1* and *Sco fabH2* ^*a*^.

Fatty acids ^*b*^	GMI1000 (%)	*Sco fabH1*/RsmH (%) ***	*Sco fabH2*/RsmH (%) *
**n-C**_**14:0**_	3.92 ± 0.23	0.68 ± 0.14	2.58 ± 0.17
***iso*-C**_**15:0**_	0	3.94 ± 0.54	0.09 ± 0.01
***anteiso*-C**_**15:0**_	0	1.13 ± 0.11	0
**n-C**_**15:0**_	0.19 ± 0.1	0.17 ± 1.59	0.33 ± 0.01
**3-OH-C**_**14:0**_	16.17 ± 2.89	8.77 ± 2.05	16.7 ± 0.59
***iso*-C**_**16:0**_	0	2.81 ± 0.21	0.57 ± 0.04
**n-C**_**16:1**_	18.12 ± 0.73	13.72 ± 0.55	15.74 ± 0.42
**n-C**_**16:0**_	25.42 ± 1.96	12.63 ± 1.59	18.56 ± 0.3
**CycleC**_**17:0**_	4.98 ± 0.49	0	2.08 ± 0.25
***iso*-C**_**17:0**_	0	21.15 ± 0.71	5.73 ± 0.27
***anteiso*-C**_**17:0**_	0	10.97 ± 0.99	0
**n-C**_**18:1**_	27.53 ± 1.69	18.49 ± 2.03	31.28 ± 2.29
**n-C**_**18:0**_	3.65 ± 0.18	5.54 ± 0.62	6.34 ± 0.20
**Total BCFAs**	0	40.0 ± 2.56	6.39 ± 0.32
**Total UFAs**	50.64 ± 2.90	32.2 ± 2.57	49.1 ± 2.96

^a^ Cells were grown in BG medium for 48 h at 30°C. The total lipids were extracted and trans-esterified to obtain fatty acid methyl esters, and the products were identified by gas chromatography-mass spectrometry. The values are percentages of total fatty acids and are the means ± standard deviations of three independent experiments. Pair-wise comparisons were made between the RsmH complementary strains and wild type strain GMI1000 by Student’s *t*-test (***P < 0.001; *P < 0.05).

^b^ n-C_14:0_, tetradecanoic acid; *iso*-C_15:0_, 13-methyl-tetradecanoic acid; *anteiso*-C_15:0_, 12-methyl-tetradecanoic acid; n-C_15:0_, pentadecanoic acid; 3-OH-C_14:0_, 3-hydroxyl tetradecanoic acid; *iso*-C_16:0_, 14-methyl-pentadecanoic acid; n-C_16:1_, *cis*-9-hexadecenoic acid; n-C_16:0_, hexadecanoic acid; CycleC_17:0_, *cis*-9,10-methylene palmitic acid, *iso*-C_17:0_, 15-methyl-hexadecanoic acid; *anteiso*-C_17:0_, 14-methyl-hexadecanoic acid; n-C_18:0_, octadecanoic acid; n-C_18:1_, *cis*-11-octadecenoic acid. UFA = unsaturated fatty acid; BCFA = branch-chain fatty acid.

### *Sco* FabH2 is active to a broad range of substrates in *in vitro* analysis

To determine KAS III activity *in vitro*, N-terminal 6× His-tagged recombinant *Sco* FabH2 was expressed in *E*. *coli* BL21(DE3) and purified by nickel chelate chromatography. Various *E*. *coli* fatty acid biosynthetic proteins, including FabD, FabB, FabG, FabA, FabI, and holo-ACP, and *Vibrio harveyi* acyl-ACP synthetase [44], were purified using the same method.

**Fig 3:**
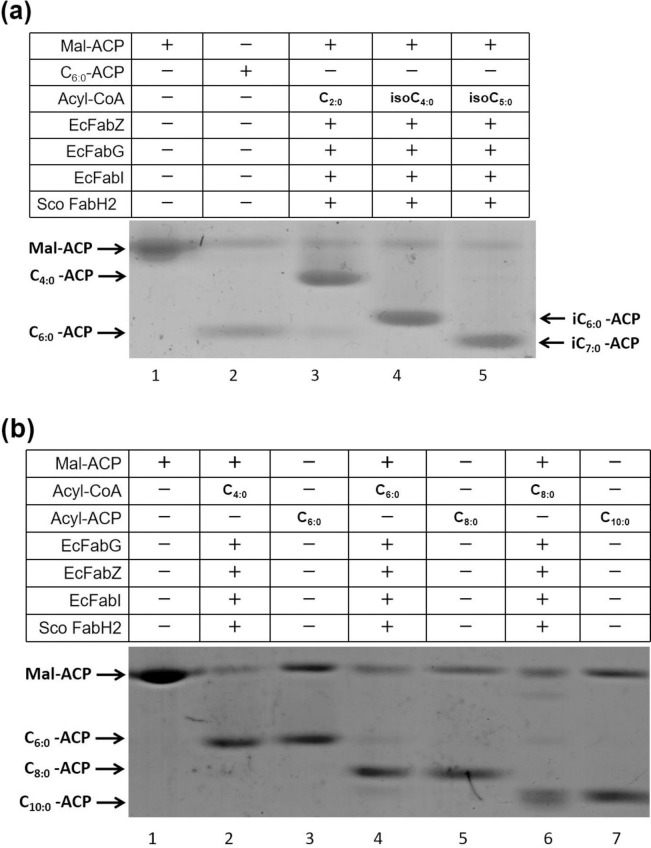
Enzymatic characterization of *Sco* FabH2 in fatty acid biosynthesis *in vitro*. (**a**) The initiation of fatty acid synthesis was reconstructed using a combination of *E*. *coli* FabZ, FabG, and FabI and *Sco* FabH2 (lanes 3–5) with NADH and NADPH as cofactors and malonyl-ACP plus acetyl-CoA (lane 3), isobutyryl-CoA (lane 4), or isovaleryl-CoA (lane 5) as substrates. The migration positions of holo-ACP (lane 1) and hexanoyl-ACP (C_6:0_-ACP, lane 2) on gel are shown. The urea concentration was 1.0 mol/L. (**b**) The initial reaction of fatty acid synthesis included *E*. *coli* FabZ, FabG, and FabI and *Sco* FabH2, NADH and NADPH as cofactors, and malonyl-ACP + butyryl-CoA (lane 2), hexanoyl-CoA (lane 4), or octacyl-CoA (lane 6) as substrates. The migration positions of holo-ACP (lane 1), hexanoyl-ACP (C_6:0_-ACP, lane 3), octacyl-ACP (C_8:0_-ACP, lane 5), and decanoyl-ACP (C_10:0_-ACP, lane 7) on gel are shown. The urea concentration was 2.5 mol/L.

The initial reaction of the fatty acid synthesis system was reconstructed based on the *E*. *coli* fatty acid synthetic enzymes mentioned. First, *Sco* FabH2 was added into the system with malonyl-ACP and acetyl-CoA as the initial substrates. The products were analyzed by conformationally sensitive gel electrophoresis. *Sco* FabH2 catalyzed the condensation of acetyl-CoA with malonyl-ACP to produce butyryl-ACP, thus exhibiting similar activity to *Ec* FabH (Fig 3A, lane 3). Interestingly, a light band of hexanoyl-ACP was detected in the products, suggesting that *Sco* FabH2 can condense butyryl-ACP with malonyl-ACP to generate long-chain acyl-ACP.

Second, the substrate specificity of *Sco* FabH2 to branched-chain acyl-CoAs was tested. When isobutyryl-CoA and isovaleryl-CoA were introduced into the fatty acid synthesis system, new products *iso*-C_6:0_-ACP (Fig 3A, lane 4) and *iso*-C_7:0_-ACP (Fig 3A, lane 5) were found in the reaction system, respectively. These results proved that *Sco* FabH2 can use branched-chain acyl-CoAs as primers to initiate fatty acid synthesis. This finding is consistent with BCFAs detected in the *Sco fabH2* complementary strain RsJR2.

Third, the activity of *Sco* FabH2 to utilize medium straight-chain acyl-CoAs as substrate was tested. *Sco* FabH2 converted butyryl (C_4:0_)-CoA to hexanoyl (C_6:0_)-ACP (Fig 3B, lane 3) and condensed hexanoyl (C_6:0_)-CoA and octanoyl (C_8:0_)-CoA with malonyl-ACP to obtain two-carbon longer acyl-ACPs (Fig 3B, lanes 5 and 7). These results demonstrate that *Sco* FabH2 can use various acyl-CoAs, including short (C_2:0_), medium straight-chain (C_4:0_ -C_8:0_), and branched-chain acyl-CoAs, as primers in fatty acid biosynthesis.

### *Sco fabH2* is not essential for growth

To further determine the physiological functions of *Sco fabH2* in *S*. *coelicolor*, we disrupted *Sco fabH2* in *S*. *coelicolor* M145 strain with an in-frame deletion. First, the upstream and downstream fragments of *Sco fabH2* were cloned and ligated into pMD19-T. Then the gene-deletion cassette was inserted into pKC1139 to obtain pMJR-8, which was further introduced into the WT M145 strain by conjugal transfer from *E*. *coli* ET12567 (pUZ8002). The apramycin-resistant exconjugants were selected and confirmed by PCR. Apramycin-sensitive colonies were screened after apramycin-free culture for three rounds of sporulation (S2a Fig in S1 File). Then the *Sco fabH2* deletion mutant strain Δ*fabH2* was obtained and verified by PCR using the primers *Sco fabH2*-5 and *Sco fabH2*-6 and by sequencing the allelic gene in the Δ*fabH2* strain. The expression of the neighboring genes *SCO6565* and *SCO6563* was confirmed using RT-qPCR (S2B Fig in S1 File). The results showed that the expressions of both genes were unaffected in the mutant. To construct a complementary strain, *Sco fabH2* was cloned and ligated into the vector pJY813 to obtain pMJR-9, in which *Sco fabH2* was expressed under a constitutive promoter kasOp* [2]. pMJR-9 was further transferred into the Δ*fabH2* strain by conjugation. Then the complementary strain C*fabH2* was selected on erythromycin- containing media and confirmed by PCR and sequencing.

**Fig 4:**
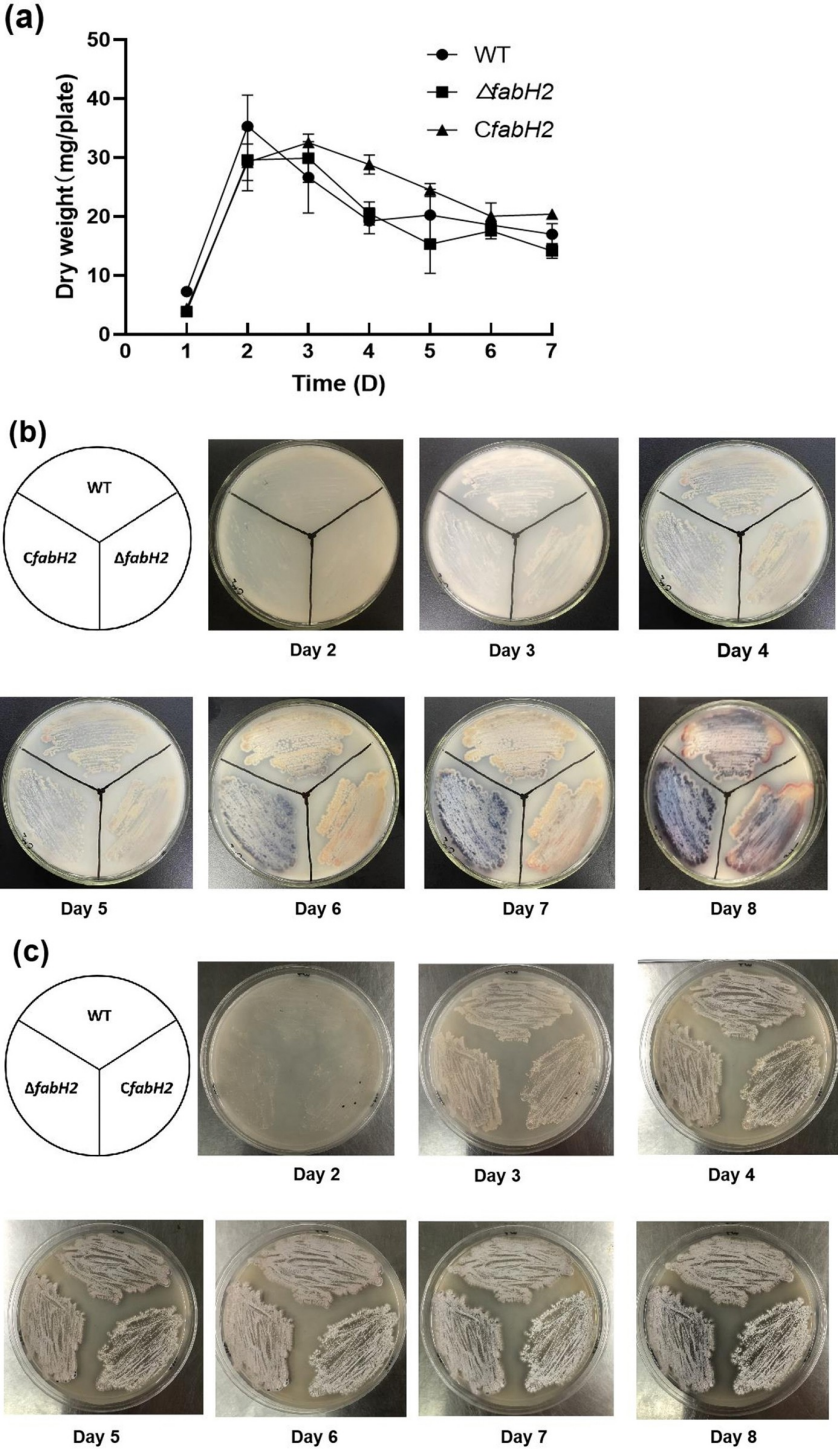
Phenotype analysis of different *S*. *coelicolor* strains. (**a**) Growth curves. Dry weight of mycelia (mg/plate) was quantified and used to represent the growth curve of different strains. Error bars indicate standard deviation of data obtained from three replicates. (**b**) Reverse side of the plates. (**c**) Front side of the plates.

The *Sco fabH1* mutant, in which the chromosomal copy of *fabH1* was replaced by *E*. *coli fabH* expressed in the plasmid, grew substantially slower than the WT strain, and a similar phenomenon was observed in the *E*. *coli fabH* deletion strain [32, 35]. We tested the growth of the different strains of *S*. *coelicolor* on YEB plates. The growth of the deletion mutant Δ*fabH2* and complementary strain C*fabH2* was comparable to that of the WT strain (Fig 4A), indicating that *Sco fabH2* is nonessential for growth. The morphological development phenotype of different strains was observed on the plate with Gauze’s synthetic medium no. 1. The phenotype of Δ*fabH2* was visibly similar to that of the WT strain after 8 d of incubation, whereas the complementary strain C*fabH2* formed white spores and accumulated blue pigment at day 6, earlier than the other two strains, suggesting that *fabH2* overexpression affected the morphological development of *S*. *coelicolor* (Fig 4B and 4C).

### *Sco fabH2* plays a minor role in fatty acid synthesis

Several reports have proved that *fabH* deletion or replacement changed fatty acid composition. Our results showed that *Sco* FabH2 actively initiates fatty acid synthesis. We hypothesize that *Sco fabH2* plays roles in fatty acid synthesis *in vivo*. To this end, we analyzed the fatty acid profiles of different *S*. *coelicolor* strains with gas chromatography–mass spectrometry. The fatty acid profile of the WT strain included BCFAs, SCFAs, and UFAs, in which BCFAs showed two forms (*iso*-BCFAs and *anteiso*-BCFAs). In *S*. *coelicolor*, BCFAs (72.58% ± 3.57%) were the predominant components, including *iso*-BCFAs (35.5% ± 2.33%) and *anteiso*-BCFAs (37.08% ± 1.24%), and the ratio of *iso*-/*anteiso*-BCFAs was 0.96. The predominant SCFA was *n*-C_16:0_ (14.73% ± 1.01%), and small amounts of UFAs (60.5% ± 113%), mainly *n*-C_17:1_, were produced in *S*. *coelicolor*. The fatty acid composition of the *Sco fabH2* deletion strain Δ*fabH2* was similar to that of the WT strain, in which the percentage of BCFAs was 76.56% ± 5.80%, and the ratio of *iso*-/*anteiso*-BCFAs was 0.96. These results demonstrated that FabH2 is not important for fatty acid synthesis in *S*. *coelicolor*. The complementary strain C*fabH2* synthesized the same fatty acids components with the other two, but the amounts of UFAs decreased, and the *anteiso*-BCFAs increased significantly to 42.21% ± 1.37%, resulting the ratio of *iso*-/*anteiso*-BCFAs declined to 0.85. All the results above indicated that *fabH2* plays minor roles in modulating fatty acid profiles (S3 Table in S1 File).

Bacterial cell membrane homeostasis is greatly affected by fatty acid composition, which play biological roles in bacterial adaption to environmental stress [19]. Mutants defective in membrane integrity exhibit increased sensitivity to detergents [45]. To test the effects of *fabH2* deletion on membrane integrity, we measured the stress response of different strains to various detergents. The results showed that Δ*fabH2* grew well as the WT strain on plates with Tween-20 and Triton X-100 (S3a & S3b Fig in S1 File). By contrast, the complementary strain C*fabH2* exhibited higher sensitivity to both detergents, indicating that *fabH2* overexpression affected membrane homeostasis (S3a &S3b Fig in S1 File). Then different strains were further tested on the plates with vancomycin and ciprofloxacin, and the results also showed that C*fabH2* exhibited more sensitive to the two antibiotics tested (S3c &S3d Fig in S1 File).

### *Sco fabH2* is not critical for ACT and RED production

**Fig 5:**
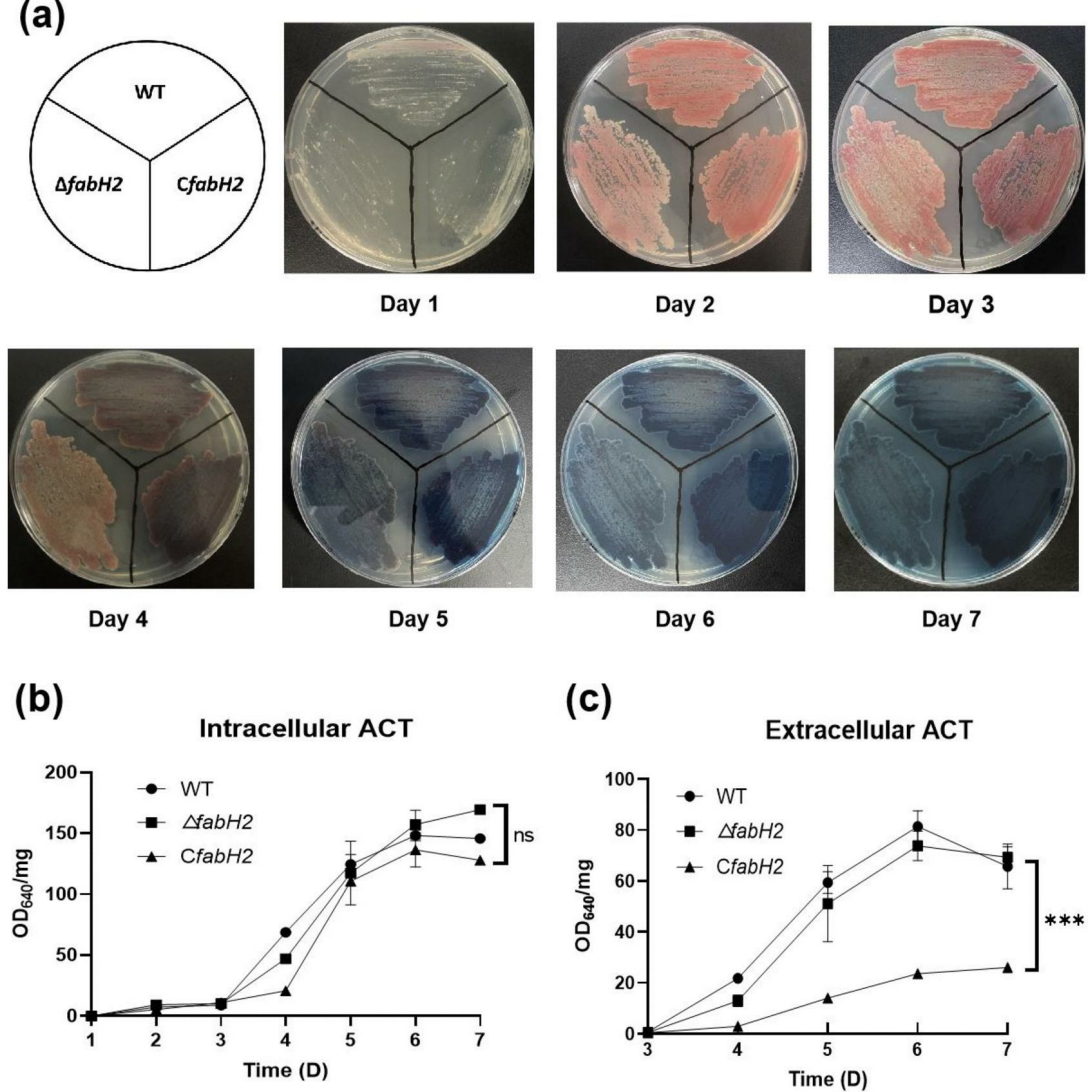
Analysis of ACT production by different strains. (**a**) Growth of different strains on YEB plates. Photographs were taken from the reverse sides of the plates. (**b**) Intracellular ACT production. (**c**) Extracellular ACT production. Intracellular ACT was quantified using only the mycelia, and extracellular ACT was quantified using only the medium. Data are expressed from three independent repeats. ns, no significant difference; ***, *P<*0.001.

*S*. *coelicolor* synthesizes two pigmented secondary metabolites as follows: ACT and RED [7]. To examine whether *fabH2* is involved in ACT and RED production, we evaluated the amounts of ACT and RED produced by different strains. On YBP plates, Δ*fabH2* showed similar phenotype as WT strain, and quantitative analysis revealed a similar trend of ACT production as WT (Fig 5). However, the complementary strain C*fabH2* decreased ACT production. In this strain, intracellular ACT was slightly lower than that in WT and Δ*fabH2*, whereas extracellular ACT was 25%–30% of WT depending on culture time (Fig 5). These results suggested that *fabH2* affects ACT production, especially under overexpression conditions. Similar results were found with RED analysis—C*fabH2* partially decreased RED production, whereas no significant differences were observed between WT and Δ*fabH2* (S4 Fig in S1 File).

## Discussion

*Streptomyces* spp. is notable for producing numerous secondary metabolites, many of which have shown promise as antibacterial, antitumor, immunosuppressant, and antifungal agents [46]. Because secondary metabolism is closely related to primary metabolism, including synthesis of fatty acids, proteins, and nucleic acids, research with primary metabolisms will pave the path for the study and application of secondary metabolites. Thus, fatty acid synthesis is studied in detail in the model strain *S*. *coelicolor*, and the series of enzymes that catalyze each step in fatty acid synthesis have been reported by various groups [47–50].

3-Ketoacyl ACP synthase III (FabH) is the key enzyme in the initiation of fatty acid synthesis. Hopwood *et al*. first reported that *S*. *coelicolor* FabH is essential for fatty acid synthesis, because *fabH* could be deleted from the chromosome to yield a viable strain but only when a second copy of *fabH* was available to complement the deletion [25]. *S*. *coelicolor* produces a broad mixture of fatty acids, predominantly BCFAs, whereas the *S*. *coelicolor fabH* deletion mutant, harboring *E*. *coli fabH* expression on a plasmid, sharply decreased BCFA content to 14%. These studies have shown that *S*. *coelicolor* FabH has greater catalytic efficiency for branched primers. By contrast, *E*. *coli* FabH only uses straight-chain acetyl-CoA as substrate. How are the remaining 14% BCFAs synthesized in the replacement mutant? One possible explanation is the presence of alternative mechanism(s) for the initiation of fatty acid biosynthesis in *S*. *coelicolor*. To this end, we first identified three additional FabH homologs that contain the same catalytic triad (Cys–His–Asn) and similar three-dimensional structures as *Sc* FabH1. In addition to *Sc fabH1*, *fabH2* (*SCO6564*) could restore the growth of *R*. *solanacearum fabH* deletion mutant under normal culture conditions, causing this strain to produce small amounts of BCFAs. This finding demonstrates that *S*. *coelicolor* FabH2 is an alternative enzyme that catalyzes the initiation of BCFAs synthesis. However, the *fabH2* complementary strain produced less amounts of BCFAs than the strain complemented with *fabH1*, indicating that FabH2 is less active than FabH1. The *fabH1* deletion caused lethality, further proving that FabH2 activity was insufficient to suppress the effect of *fabH1* deletion.

The activities of FabH2 were further confirmed using *in vitro* assays, which demonstrated that FabH2 condensed different substrates, such as short-chain-, medium-chain-, and branched-chain acyl-CoAs, with malonyl-ACP to initiate fatty acid biosynthesis. Unfortunately, when we probed the substrate specificity of FabH2 by monitoring the rate of oxidation of NADPH at 340 nm, the activities of FabH2 to the primers were not detectable *in vitro* [26], which confirmed that the catalytic activity of FabH2 was relatively weak. The finding is consistent with the low content of BCFAs in the complementary strain.

The deletion of *S*. *coelicolor fabH2* did not affect bacterial growth and produced large amounts of BCFAs, demonstrating that *fabH2* is nonessential for growth and BCFAs synthesis. Then we attempted to construct a replacement mutant in which *fabH1* was deleted in the chromosome by introducing a *fabH2*-encoded plasmid (pMJR-9). However, we failed and no mutant was selected. The most reasonable explanation is that the biological functions of *fabH1* in fatty acid biosynthesis cannot be replaced by *fabH2* because of its low catalytic activities, even when overexpressed. Another possibility is that FabH2 has biological function in secondary metabolism, e.g., the FabH homolog RedP plays a role in undecylprodiginine synthesis in *S*. *coelicolor* [8]. Therefore, we tested the production of ACT and RED in the *fabH2* deletion mutant. We found no significant differences in ACT and RED production between the WT strain and the *fabH2* deletion mutant, indicating that FabH2 is dispensable for ACT and RED synthesis. To our surprise, the complementary strain C*fabH2* produced much less ACT than the WT and *fabH2* deletion strain. The reason for this finding is unclear. We hypothesize that *fabH2* overexpression, to some extent, caused minor changes inside *S*. *coelicolor*, which further affected ACT synthesis. Similar results were observed when testing membrane permeability. The C*fabH2* strain became more sensitive to detergents and antibiotics, which may be caused by the variation in the ratio of *iso*-/*anteiso*-BCFAs, although the whole fatty acid profiles showed no significant changes.

**Fig 6:**
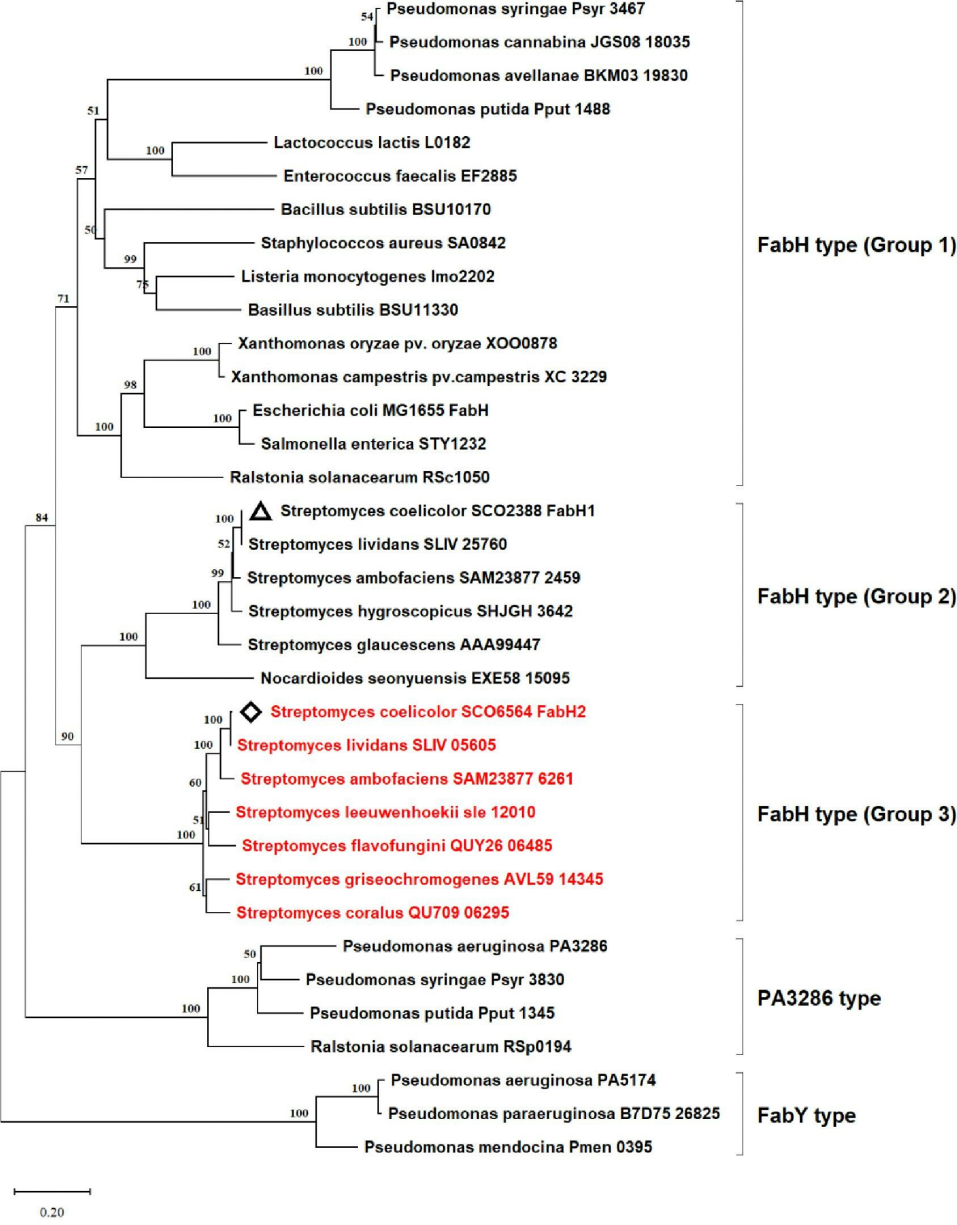
Maximum-likelihood phylogenetic analysis of KAS III from different bacteria. The maximum-likelihood tree was created using MEGA 11. Proteins are shown with their KEGG accession numbers clustered into five major groups. The most common FabHs reported clustered in group 1, *S*. *coelicolor* FabH1 (marked with delta) clustered in group 2, and *S*. *coelicolor* FabH2 (marked with diamond) clustered in group 3.

Although the FAS II mechanism is relatively conserved in bacteria, variations in the initiation reactions have been identified in distinct bacteria—the KAS III pathway [24, 26] and the MAD pathway [31, 33]. Using the maximum-likelihood algorithm in the software MEGA 11 [51], a phylogenetic analysis of KAS III from different bacteria was constructed (Fig 6). The results showed that KAS III could be further classified into FabH type, PA3286 type, and FabY type, which differed in the types of substrates used and had structural differences [27, 28]. FabH type could be further classified into three groups. *E*. *coli* FabH, two *B*. *subtilis* FabHs (BSU11330 and BSU10170), *Xcc* FabH (XC3229), *Xoo* FabH (XOO0878), etc., were clustered in group 1, confirming that FabH type (group 1) was widely distributed in gram-positive and gram-negative bacteria. FabH type (group 2) seemed to be conserved in actinomycetes, of which *S*. *coelicolor* FabH1 (SCO2388) and *S*. *glaucescens* FabH (AAA99447) have been intensively studied. *S*. *coelicolor* FabH2 (SCO6564) belongs to FabH type (group 3), which is ubiquitous in *Streptomyces* spp. A comparative analysis of the *S*. *coelicolor* FabH2 sequence extended the presence of its homologs to *S*. *lividans*, *S*. *ambofaciens*, *S*. *coralus*, etc (S5 Fig in S1 File). The top 100 homologs had protein identities of 80.8% –99.7% in the Kyoto Encyclopedia of Genes and Genomes database.

Overall, *S*. *coelicolor SCO6564* encodes an active KAS III, FabH2, which can catalyze the initiation of fatty acid synthesis with different substrates, but FabH2 cannot replace the conserved FabH1, because it has weak activity. *S*. *coelicolor* FabH2 represents a novel group of FabH type KAS III, which mainly presents in *Streptomyces* spp. and its biological functions need further study.

## Supporting information

S1 File Supplementary files table & figure. (ZIP)

S1 Raw images (PDF)

## References

[1] Rodrigo CB, Augusto ZA, Beatriz RV, Adelfo EL, Romina RS, Sergio S. An overview of the two-component system GarR/GarS role on antibiotic production in *Streptomyces coelicolor*. *Appl Microbiol Biot* 2024, 108: 306. 10.1007/s00253-024-13136-z PMC11043171 38656376

[2] Zhao MX, Yang ZY, Li XY, Liu YQ, Zhang YY, Zhang MQ, et al. Development of integrated vectors with strong constitutive promoters for high-yield antibiotic production in mangrove-derived *Streptomyces*. *Marine drugs* 2024, 22: 94. 38393065 10.3390/md22020094 PMC10890193

[3] Somasekhara D, Varalakshmi KN. Analysis of the anticancer cechanism of OR3 pigment from *Streptomyces coelicolor* JUACT03 against the human hepatoma cell line using a proteomic approach. *Cell Biochemistry and Biophysics* 2024, 82(2):1061–1077. 38578403 10.1007/s12013-024-01258-0

[4] Bikash B, Soheila M, Vilja S, Keshav T, Amir A, Keith Y, et al. Co-factor independent oxidases ncnN and actVA-3 are involved in the dimerization of benzoisochromanequinone antibiotics in naphthocyclinone and actinorhodin biosynthesis. *FEMS Microbiology Letters* 2023, 370, 1–10. 10.1093/femsle/fnad123 PMC10697411 37989784

[5] Seipke RF, Kaltenpoth M, Hutchings MI. *Streptomycesas* symbionts: an emerging and widespread theme? *FEMS Microbiology Reviews* 2012, 36, 862–876. 22091965 10.1111/j.1574-6976.2011.00313.x

[6] Bi Y, An H, Chi Z, Xu Z, Deng Y, Ren Y, et al. The acetyltransferase SCO0988 controls positively specialized metabolism and morphological differentiation in the model strains *Streptomyces coelicolor* and *Streptomyces lividans*. *Frontiers in microbiology* 2024, 15. 10.3389/fmicb.2024.1366336 PMC11303876 39113837

[7] Kanchanabanca C, Hosaka T, Kojima M. High-intensity green light potentially activates the actinorhodin biosynthetic pathway in *Streptomyces coelicolor* A3(2). *Archives of Microbiology* 2023, 206. 10.1007/s00203-023-03730-6 38038757

[8] Singh R, Mo S, Florova G, Reynolds KA. *Streptomyces coelicolor* RedP and FabH enzymes, initiating undecylprodiginine and fatty acid biosynthesis, exhibit distinct acyl-CoA and malonyl-acyl carrier protein substrate specificities. *FEMS Microbiol Lett* 2012, 328, 32–38. 22136753 10.1111/j.1574-6968.2011.02474.x

[9] Cronan JE, Thomas J. Bacterial fatty acid synthesis and its relationships with polyketide synthetic pathways. *Methods Enzymol* 2009, 459, 395–433. doi: 10.1016/S0076-6879(09)04617-5 19362649 PMC4095770

[10] Zimhony O, Vilcheze C, Jacobs WR Jr. Characterization of *Mycobacterium smegmatis* expressing the *Mycobacterium tuberculosis* fatty acid synthase I (*fas1*) gene *J Bacteriol* 2004, 186, 4051–4055. 15205406 10.1128/JB.186.13.4051-4055.2004 PMC421601

[11] Cronan JE. The acyl carrier proteins of lipid synthesis are busy having other affairs. *Biochem J* 2023, 480, 855–873. doi: 10.1042/BCJ20230161 37345808

[12] Cronan JE. Advances in synthesis of biotin and assembly of lipoic acid. *Curr Opin Chem Biol* 2018, 47, 60–66. doi: 10.1016/j.cbpa.2018.08.004 30236800 PMC6289770

[13] Huang YH, Lin JS, Ma JC, Wang HH. Functional characterization of triclosan-resistant enoyl-acyl-carrier protein reductase (FabV) in *Pseudomonas aeruginosa*. *Frontiers in microbiology* 2016, 7. 10.3389/fmicb.2016.01903 PMC5126088 27965638

[14] Yan MF, Yu YH, Luo LZ, Huang M, Zhang YY, Su JT, et al. 3-Ketoacyl-ACP synthase III FabH1 is essential for branched-chain DSF family signals in *Xanthomonas oryzae* pv. *oryzae*. *Phytopathology Research* 2023, 5: 26.

[15] Hu Z, Dong HJ, Ma JC, Yu YH, Li KH, Guo QQ, et al. Novel *Xanthomonas campestris* long-chain-specific 3-oxoacyl-acyl carrier protein reductase involved in diffusible signal factor synthesis. *MBio* 2018, 9: e00596–00518. 29739899 10.1128/mBio.00596-18 PMC5941067

[16] Yao J, Rock CO. Bacterial fatty acid metabolism in modern antibiotic discovery. *Biochimica et biophysica acta Molecular and cell biology of lipids* 2017, 1862, 1300–1309 doi: 10.1016/j.bbalip.2016.09.014 27668701 PMC5364071

[17] Cheng JL, Ma JC, Lin JS, Fan ZC, Cronan JE, Wang HH. Only one of the five *Ralstonia solanacearum* long-chain 3-ketoacyl-acyl carrier protein synthase homologues functions in fatty acid synthesis. *Applied and environmental microbiology* 2012, 78, 1563–1573. 22194290 10.1128/AEM.07335-11 PMC3294497

[18] Parsons JB, Rock CO. Bacterial lipids: metabolism and membrane homeostasis *Prog Lipid Res* 2013, 52, 249–276. doi: 10.1016/j.plipres.2013.02.002 23500459 PMC3665635

[19] Zhang YM, Rock CO Membrane lipid homeostasis in bacteria *Nat Rev Microbiol* 2008, 6, 222–233. doi: 10.1038/nrmicro1839 18264115

[20] Tsay JT, Oh W, Larson TJ, Jackowski S, Rock CO. Isolation and characterization of the beta-ketoacyl-acyl carrier protein synthase III gene (*fabH*) from *Escherichia coli* K-12. *J Biol Chem* 1992, 267, 6807–6814. 1551888

[21] Rock CO, Cronan JE. *Escherichia coli* as a model for the regulation of dissociable (type II) fatty acid biosynthesis. *Biochim Biophys Acta* 1996, 1302, 1–16. 8695652 10.1016/0005-2760(96)00056-2

[22] Mao YH, Ma JC, Li F, Hu Z, Wang HH. *Ralstonia solanacearum* RSp0194 encodes a novel 3-keto-acyl carrier protein synthase III. *Plosone* 2015, 10(8): e0136261. 10.1371/journal.pone.0136261 PMC4549310 26305336

[23] Choi KH, Health RJ, Rock CO. Beta-ketoacyl-acyl carrier protein synthase III (FabH) is a determining factor in branched-chain fatty acid biosynthesis. *J Bacteriol* 2000, 182, 365–370. doi: 10.1128/JB.182.2.365-370.2000 10629181 PMC94284

[24] Radka CD, Rock CO. Crystal structures of the fatty acid biosynthesis initiation enzymes in *Bacillus subtilis*. *J Struct Biol* 2024, 216. 10.1016/j.jsb.2024.108065 PMC10939784 38310992

[25] Revill WP, Bibb MJ, Scheu AK, Kieser HJ, Hopwood DA. Beta-ketoacyl acyl carrier protein synthase III (FabH) is essential for fatty acid biosynthesis in *Streptomyces coelicolor* A3(2). *J Bacteriol* 2001, 183, 3526–3530. 11344162 10.1128/JB.183.11.3526-3530.2001 PMC99652

[26] Yu YH, Hu Z, Dong HJ, Ma JC, Wang HH. *Xanthomonas campestris* FabH is required for branched-chain fatty acid and DSF-family quorum sensing signal biosynthesis. *Sci Rep* 2016, 6, 32811 27595587 10.1038/srep32811 PMC5011732

[27] Yuan Y, Sachdeva M, Leeds JA, Meredith TC. Fatty acid biosynthesis in *Pseudomonas aeruginosa* is initiated by the FabY class of beta-ketoacyl acyl carrier protein synthases. *J Bacteriol* 2012, 194, 5171–5184. 22753059 10.1128/JB.00792-12 PMC3457228

[28] Yuan Y, Leeds JA, Meredith TC. *Pseudomonas aeruginosa* directly shunts beta-oxidation degradation intermediates into *de novo* fatty acid biosynthesis. *J Bacteriol* 2012, 194, 5185–5196. 22753057 10.1128/JB.00860-12 PMC3457203

[29] Guo QQ, Zhang WB, Zhang C, Song YL, Liao YL, Ma JC, et al. Characterization of 3-oxacyl-acyl carrier protein reductase homolog genes in *Pseudomonas aeruginosa* PAO1. Front Microbiol 2019, 10:1028. 31231314 10.3389/fmicb.2019.01028 PMC6558427

[30] Guo QQ, Su JT, Liao YL, Yin Y, Luo LZ, Weng XS, et al. An atypical 3-ketoacyl ACP synthase III required for acyl homoserine lactone synthesis in *Pseudomonas syringae* pv. *syringae* B728a. *App Environ Microbiol* 2024, 90(3): e0225623. 10.1128/aem.02256-23 PMC10952384 38415624

[31] Guo QQ, Zhang C, Dong HJ, Cronan JE, Wang HH. Diversity in fatty acid elongation enzymes: The FabB long-chain β-ketoacyl-ACP synthase I initiates fatty acid synthesis in *Pseudomonas putida* F1. *J Biol Chem* 2024, 300(2), 105600. 38335573 10.1016/j.jbc.2023.105600 PMC10869286

[32] McNaught KJ, Kuatsjah E, Zahn M, Prates ÉT, Shao H, Bentley GJ, et al. Initiation of fatty acid biosynthesis in *Pseudomonas putida* KT2440 *Metab Eng* 2023, 76, 193–203. 36796578 10.1016/j.ymben.2023.02.006

[33] Sanyal R, Singh V, Harinarayanan RA. Novel gene contributing to the initiation of fatty acid biosynthesis in *Escherichia coli*. *J Bacteriol* 2019, 201, e00354–00319. 31331975 10.1128/JB.00354-19 PMC6755751

[34] Whaley SG, Radka CD, Subramanian C, Frank MW, Rock CO. Malonyl-acyl carrier protein decarboxylase activity promotes fatty acid and cell envelope biosynthesis in *Proteobacteria*. *J Biol Chem* 2021, 297, 101434. 34801557 10.1016/j.jbc.2021.101434 PMC8666670

[35] Yu YH, Ma JR, Miao XY, Wang HH. Biological function research of 3-ketoacyl ACP synthase Ⅲ from different bacteria. *Prog Biochem Biophys*, 2016, 43, 1004–1012.

[36] Li YL, Florova G, Reynolds KA. Alteration of the fatty acid profile of *Streptomyces coelicolor* by replacement of the initiation enzyme 3-ketoacyl acyl carrier protein synthase III (FabH). *J Bacteriology* 2005, 187(11):3795–3799. 10.1128/JB.187.11.3795-3799.2005 PMC1112031 15901703

[37] Lu T, Cao Q, Pang X H, Xia YZ, Xun LY, Liu HW. Sulfane sulfur-activated actinorhodin production and sporulation is maintained by a natural gene circuit in *Streptomyces coelicolor*. *Microb Biotechnol* 2020, 13(6): 1917–1932. 32776457 10.1111/1751-7915.13637 PMC7533328

[38] Atlas RM. Handbook of Microbiological Media CRC Press 1993.

[39] Khan SR, Gaine J, Roop RMII, Farrand SK. Broad-host-range expression vectors with tightly regulated promoters and their use to examine the influence of TraR and TraM expression on Ti plasmid quorum sensing. *Appl Environ Microbiol* 2008, 74, 5053–5062. doi: 10.1128/AEM.01098-08 18606801 PMC2519271

[40] Yu YH, Chen C, Ma JR, Zhang YY, Yan MF, Zhang WB, et al. The FabA-FabB pathway is not essential for unsaturated fatty acid synthesis but modulates diffusible signal factor synthesis in *Xanthomonas campestris* pv. *campestris*. *Mol Plant Microbe Interact* 2023, 36, 119–130. 36515967 10.1094/MPMI-09-22-0182-R

[41] Shang GD, Dai JL, Wang YG. Construction and physiological studies on a stable bioengineered strain of shengjimycin. *J Antibiot (Tokyo)* 2001, 54(1): 66–73. doi: 10.7164/antibiotics.54.66 11269716

[42] Yu YH, Ma JR, Guo QQ, Ma JC, Wang HH. A novel 3-oxoacyl-ACP reductase (FabG3) is involved in the xanthomonadin biosynthesis of *Xanthomonas campestris* pv. *campestris*. *Mol Plant Pathol* 2019, 20(12):1696–1709. 31560825 10.1111/mpp.12871 PMC6859482

[43] Kieser T, Bibb MJ, Buttner MJ, Chater KF, Hopwood DA. Practical streptomyces genetics. Norwich, UK: John Innes Foundation 2000.

[44] Qiu X, Janson CA, Smith WW, Head M, Lonsdale J, Konstantinidis AK. Refined structures of beta-ketoacyl-acyl carrier protein synthase III. *J Mol Biol* 2001, 307, 341–356. doi: 10.1006/jmbi.2000.4457 11243824

[45] Singh P, Verma RK, Chatterjee S. The diffusible signal factor synthase, RpfF, in *Xanthomonas oryzae* pv. *oryzae* is required for the maintenance of membrane integrity and virulence. *Mol Plant Pathol* 2022, 23, 118–132. 34704368 10.1111/mpp.13148 PMC8659556

[46] Lei YK, Asamizu S, Ishizuka T, Onaka H. Regulation of multidrug efflux pumps by TetR family transcriptional repressor negatively affects secondary metabolism in *Streptomyces coelicolor* A3(2). *Appl Environ Microbiol* 2023, 89(3):e0182222. 36790176 10.1128/aem.01822-22 PMC10056966

[47] Singh R, Reynolds KA. Characterization of FabG and FabI of the *Streptomyces coelicolor* dissociated fatty acid synthase. *Chembiochem* 2015, 16, 631–640. 25662938 10.1002/cbic.201402670

[48] Tang Y, Lee HY, Kim CY, Mathews I, Khosla C. Structural and functional studies on SCO1815: a beta-ketoacyl-acyl carrier protein reductase from *Streptomyces coelicolor* A3(2). *Biochemistry* 2006, 45, 14085–14093. 17115703 10.1021/bi061187v

[49] Revill WP, Bibb MJ, Hopwood DA. Relationships between fatty acid and polyketide synthases from *Streptomyces coelicolor* A3(2): characterization of the fatty acid synthase acyl carrier protein. *J Bacteriol* 1996, 178, 5660–5667. 8824610 10.1128/jb.178.19.5660-5667.1996 PMC178404

[50] Singh R, Reynolds KA. Identification and characterization of FabA from the type II fatty acid synthase of *Streptomyces coelicolor*. *J Nat Prod* 2016, 79, 240–243. 26731437 10.1021/acs.jnatprod.5b00560

[51] Tamura K, Stecher G, Kumar S. MEGA 11: molecular evolutionary genetics analysis version 11. *Mol Biol Evol* 2021, 38: 3022–3027. doi: 10.1093/molbev/msab120 33892491 PMC8233496

