# Supplementary material for: SCO6564, a novel 3-ketoacyl acyl carrier protein synthase III, contributes in fatty acid synthesis in Streptomyces coelicolor
Source: PLoS One. 2025 Feb 6;20(2):e0318258. doi: 10.1371/journal.pone.0318258 (PMC11801535; doi:10.1371/journal.pone.0318258)
Supplement: S1 File — (ZIP) [file pone.0318258.s001.zip › Supplimentary files table & figure.docx]

**Supplementary Tables**

**Table S1. Bacterial strains and plasmids used in this study**

| **Bacterial strains** | **Relevant characteristics*^a^*** | **Source** |
| --- | --- | --- |
| ***E. coli*** |  |  |
| **DH-5α** | F^-^ *deoR* *endA1 gyrA96 hsdR17*(r_K_^-^m_K_^+^) *recA1* *relA1* *supE*44 *thi-1* Δ(*lacZYA-argF*)*U*169(φ80*lacZ*ΔM15) | Lab stock |
| **BL21(DE3)** | F^-^ *dcm* *omp T* *hsdS*(r_B_^-^m_B_^-^) *gal* (λDE3) | Lab stock |
| **S17-1** | Tp^r^ Sm^r^ *recA* *thi* *pro* *hsdR* (RP4-2 Tc::Mu Km::Tn*7*), λ*pir* | Lab stock |
| ***E. coli* ET12567** | Cm^R^, Km^R^, Methylation-defective strain used in *E.coli-Streptomyses* conjugation | Lab stock |
|  |  |  |
| ***R. solanacearum*** | |  |
| **GMI1000** | Cm^r^, Wild-type strain | ATCC |
| **RsmH** | Cm^r^, GMI1000 Δ*fabH* | 32 |
| **RsJR1** | Cm^r^, Km^r^, GMI1000 Δ*fabH/* pSRK- *Sco* *fabH1* | This work |
| **RsJR2** | Cm^r^, Km^r^, GMI1000 Δ*fabH/* pSRK- *Sco* *fabH2* | This work |
| **RsJR3** | Cm^r^, Km^r^, GMI1000 Δ*fabH/* pSRK- *Sco* *fabH3* | This work |
| **RsJR4** | Cm^r^, Km^r^, GMI1000 Δ*fabH/* pSRK- *Sco* *fabH4* | This work |
|  |  |  |
| ***S*. *coelicolor*** |  |  |
| **M145** | Wild type | Lab stock |
| **Δ*fabH2*** | *fabH2* deletion in the wild type strain | This work |
| **C*fabH2*** | Em^r^, Δ*fabH2* / pJY-*Sco fabH2* | This study |
|  |  |  |
| **Plasmids** |  |  |
| **pET-28(b)** | Km^r^, T7 promoter-based expression vector | Novagen |
| **pMD19-T** | Amp^r^, TA cloning vector | Takara |
| **pSRK-Km** | Km^r^, broad-host-range expression vector containing *lac* promoter and *lacI*^q^, *lacZ*α^+^ | ^39^ |
| **pKC1139** | Am^R^, *E. coli-Streptomyces* shuttle plasmid, temperature sensitive, *oriT*(RP4), *ori* (pSG5) | Lab stock |
| **pJY813** | Amp^R^, Erm^R^, *E. coli-Streptomyces* shuttle plasmid, *int-attP* (φC31), *PkasOp** promoter, *To* terminator | Lab stock |
| **pMJR-1** | Km^r^, *Sco fabH1* cloned into plasmid pSRK-Km | This study |
| **pMJR-2** | Km^r^, *Sco fabH2* cloned into plasmid pSRK-Km | This study |
| **pMJR-3** | Km^r^, *Sco fabH3* cloned into plasmid pSRK-Km | This study |
| **pMJR-4** | Km^r^, *Sco fabH4* cloned into plasmid pSRK-Km | This study |
| **pMJR-5** | Km^r^, *Sco fabH2* cloned into plasmid pET-28(b) | This study |
| **pMJR-6** | Amp^r^, the upstream fragment of *Sco fabH2* cloned into pMD19-T | This study |
| **pMJR-7** | Amp^r^, the upstream and downstream fragments of *Sco fabH2* (Δ*fabH2*) cloned into pMD19-T | This study |
| **pMJR-8** | Am^R^, Δ*fabH2* inserted into pKC1139 | This study |
| **pMJR-9** | Amp^R^, Erm^R^, *Sco fabH2* gene inserted to pJY813 | This study |

**Note:** Cm, chloramphenicol; Km, kanamycin; Erm, erythromycin; Amp, ampicillin; Am, apramycin

**Table S2. Sequences of the PCR primers used in this work**

| **Primer name** | **Primer sequence (5’ to 3’)** | **Digestion sites ^a^** |
| --- | --- | --- |
| **Gene amplification** | | |
| *Sco fabH1* UP | AATTGCGCATATGTCGAAGATCAAGCCCAG | *Nde* Ⅰ |
| *Sco fabH1* DN | AATTAAGCTTACGGAGTGCCTAGGGGAG | *Hin*d Ⅲ |
| *Sco fabH2* UP | AATACGGCATATGCACCAAGGCTCCCGC | *Nde* Ⅰ |
| *Sco fabH2* DN | AATTAAGCTTGTAGGCCACGTCACACC | *Hin*d Ⅲ |
| *Sco fabH3* UP | AATACGGCATATGGCCCGGGGCGCGGGGC | *Nde* Ⅰ |
| *Sco fabH3* DN | AATTAAGCTTGGTGCTCCTTACGGAAGTTCG | *Hin*d Ⅲ |
| *Sco fabH4* UP | AATACGGCATATGAGCGCCGCGCGCGGC | *Nde* Ⅰ |
| *Sco fabH4* DN | AATTAAGCTTTCGGTCCGGTCAGCTCC | *Hin*d Ⅲ |
| *Sco fabH2*-P1 | GGAATTCCATATGCACCAAGGCTCCCGC | *Nde* Ⅰ |
| *Sco fabH2*-P2 | AGAGATCTTCAGGGGCAGCGGACGAC | *Bgl* II |
| **Gene deletion** | | |
| *Sco fabH2*-1 | AATTAAGCTTCAGTACTGGCTGATCGTCCT | *Hin*d Ⅲ |
| *Sco fabH2*-2 | GACGTTCACTGCAGCATGGGTGGAGTCCTCGCT | *Pst* I |
| *Sco fabH2*-3 | GACTCCACCCATGCTGCAGTGAACGTCCCGGACCGTC | *Pst* I |
| *Sco fabH2*-4 | AATTGAATTCAGTGGCTGGTTGATGTGC | *Eco* RI |
| *Sco fabH2*-5 | TTGTCGGCGTGGCTTGCTTTC |  |
| *Sco fabH2*-6 | CCTGGTCGTCGTGTCCTTCTAC |  |
| **RT-qPCR analysis** | | |
| *hrdB*-rt-f | CGCCGAGTCCGTCTCTGTCA |  |
| *hrdB*-rt-r | GCTCTGCGGCACTGACCATC |  |
| SCO6563-rt-f | TCGGCGTGCTGCTGGTA |  |
| SCO6563-rt-f | TGGAGGTGTCGGAGAAGAA |  |
| SCO6565-rt-f | TGCGGGACCGTGTCTAC |  |
| SCO6565r-rt-r | CCTCCTCCGTCGGCTCG |  |

*^a^* underlined nucleotide sequences are digestion sites of restriction endonuclease.

**Table S3. Fatty acid compositions of different *S. coelicolor* strains *^a^***

| **Fatty Acids *^b^*** | **WT (%)** | **Δ*fabH2* (%)** | **C*fabH2* (%)** |
| --- | --- | --- | --- |
| ***iso*-C_13:0_** | 0.46 ± 0.24 | 0.38 ± 0.12 | 0.28 ± 0.02 |
| ***iso*-C_14:0_** | 2.03 ± 0.01 | 2.52 ± 0.31 | 2.01 ± 0.14 |
| **n-C_14:0_** | 0.96 ± 0.3 | 0.23 ± 0.2 | 0.54 ± 0.09 |
| ***iso*-C_15:0_** | 9.35 ± 0.9 | 9.41 ± 0.76 | 9.58 ± 0.63 |
| ***anteiso*-C_15:0_** | 20.5 ± 1.02 | 21.16 ± 0.03 | 22.6 ± 0.78 |
| **n-C_15:0_** | 3.01 ± 0.26 | 2.82 ± 0.51 | 4.23 ± 0.84 |
| ***iso*-C_16:0_** | 15.79 ± 0.52 | 17.74 ± 1.24 | 16.62 ± 1.24 |
| **n-C_16:1_** | 1.32 ± 0.23 | 0.8 ± 0.12 | 0.53 ± 0.05 |
| **n-C_16:0_** | 14.73 ± 1.01 | 10.37 ± 1.77 | 8.74 ± 0.67 |
| **n-C_17:1_** | 2.67 ± 0.27 | 2.39 ± 0.08 | 1.87 ± 1.1 |
| ***iso*-C_17:0_** | 7.36 ± 0.42 | 6.87 ± 0.38 | 7.12 ± 0.15 |
| ***anteiso*-C_17:0_** | 16.58 ± 0.22 | 17.9 ± 2.79 | 19.61 ± 0.59 |
| **Cyc-C_17:0_** | 1.83 ± 0.53 | 1.51 ± 0.32 | 0.87 ± 0.33 |
| **n-C_17:0_** | 3.42 ± 0.97 | 3.31 ± 2.89 | 2.68 ± 2.48 |
| ***iso*-C_18:0_** | 0.52 ± 0.25 | 0.57 ± 0.17 | 0.58 ± 0.16 |
| **n-C_18:1_** | 0.24 ± 0.1 | 0.25 ± 0.09 | 0.28 ± 0.06 |
| **n-C_18:0_** | 1.53 ± 0.63 | 1.77 ± 0.59 | 1.82 ± 0.53 |
|  |  |  |  |
| **UFAs** | 6.05 ± 1.13 | 4.94 ± 0.62 | 3.56 ± 1.54* |
| ***iso*-BCFAs** | 35.5 ± 2.33 | 37.49 ± 2.98 | 36.2 ± 2.35 |
| ***anteiso*-BCFAs** | 37.08 ± 1.24 | 39.07 ± 2.82 | 42.21 ± 1.37** |
| **BCFAs** | 72.58 ± 3.57 | 76.56 ± 5.80 | 78.41 ± 3.71 |
| ***iso*-/*anteiso*-** | **0.96** | **0.96** | **0.85** |

***^a^*** Different *S. coelicolor* strains were grown in YBP medium for 3 days at 30°C. The total lipids were extracted and trans-esterified to obtain fatty acid methyl esters, and the products were identified by gas chromatography-mass spectrometry. The values are percentages of total fatty acids and are the means ± standard deviations of three independent experiments. On asterisk (*) indicates *P* < 0.05, two (**) *P* < 0.001, assessed by one-way analysis of variance comparing with WT.

***^b^*** *iso*-C_13:0_, 11-methyl-dodecanoic acid; *iso*-C_14:0_, 12-methyl-tridecanoic acid; n-C_14:0_, tetradecanoic acid; *iso*-C_15:0_, 13-methyl-tetradecanoic acid; *anteiso*-C_15:0_, 12-methyl- tetradecanoic acid; n-C_15:0_, pentadecanoic acid; *iso*-C_16:0_, 14-methyl-pentadecanoic acid; n-C_16:1_, *cis*-9-hexadecenoic acid; n-C_16:0_, hexadecanoic acid; *iso*-C_17:0_, 15-methyl -hexadecanoic acid; *anteiso*-C_17:0_, 14-methyl-hexadecanoic acid; *iso*-C_18:0_, 16-methyl- heptadecanoic acid; n-C_18:1_, *cis*-11-octadecenoic acid; n-C_18:0_, octadecanoic acid. UFA indicates unsaturated fatty acid; BCFA indicates branch-chain fatty acid.


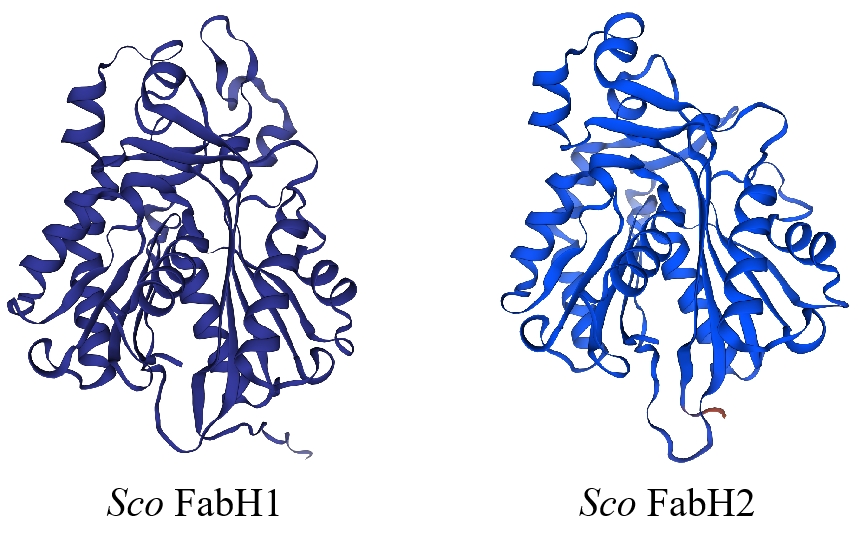


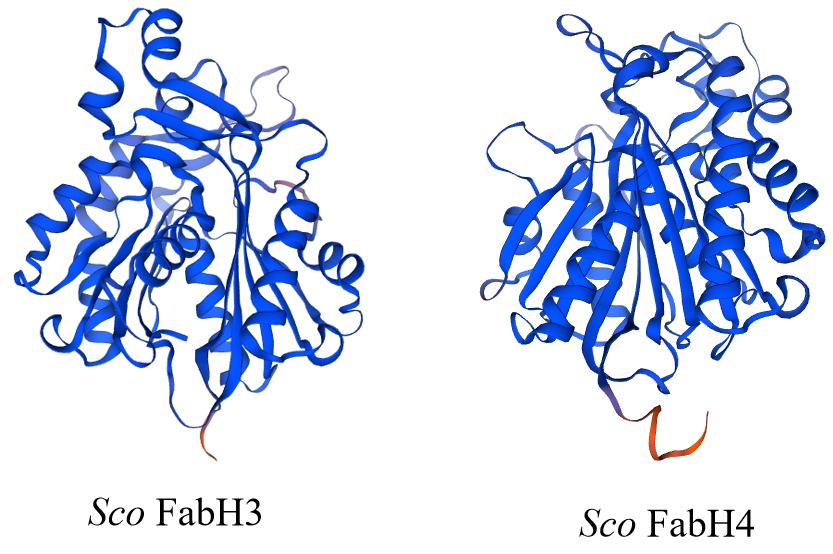


Figure S1 The three-dimensional structures of *Sco* FabHs prediction using the online SWISS-MODEL (https://swissmodel.expasy.org).


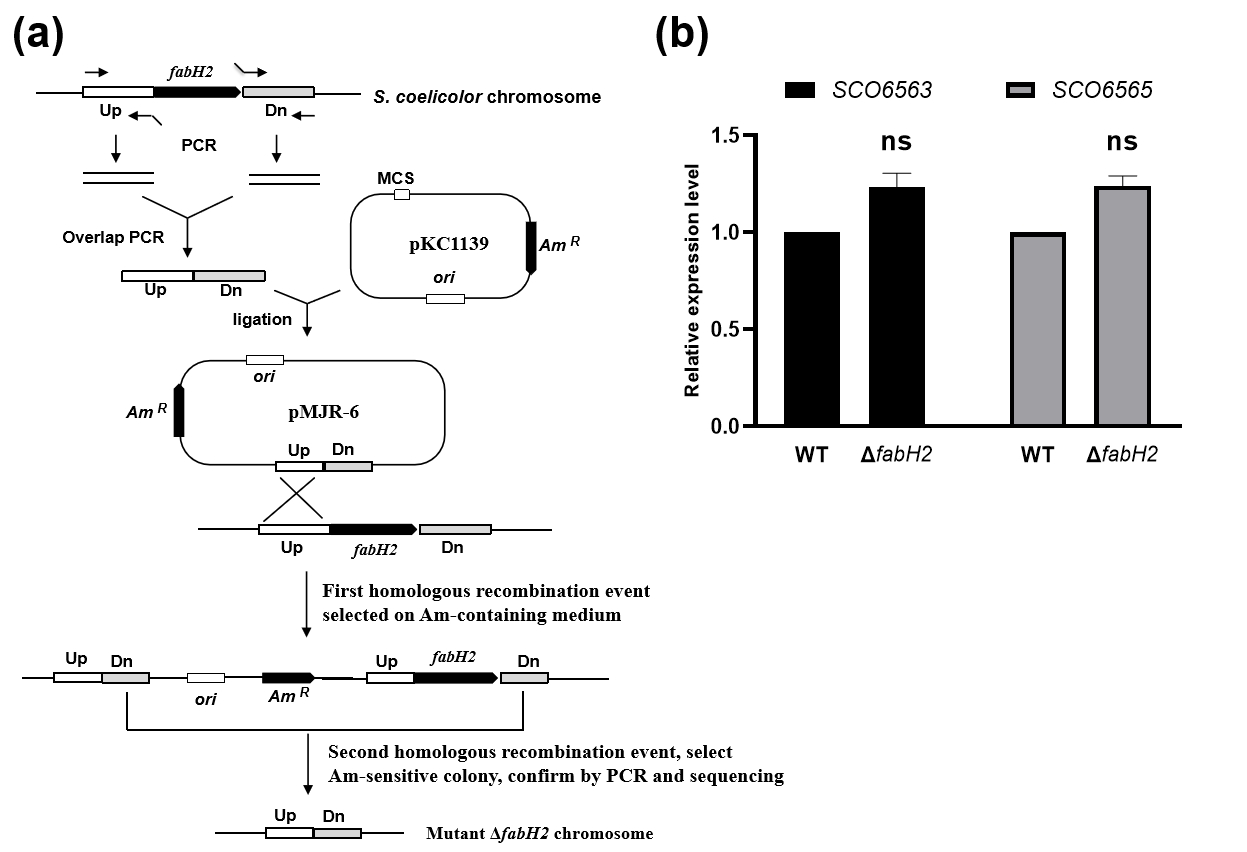


Figure S2 (a) The in-frame deletion mutant Δ*fabH2* construction strategy; (b) RT-qPCR analysis of the *fabH2* neighboring genes *SCO6563* and *SCO6565*.


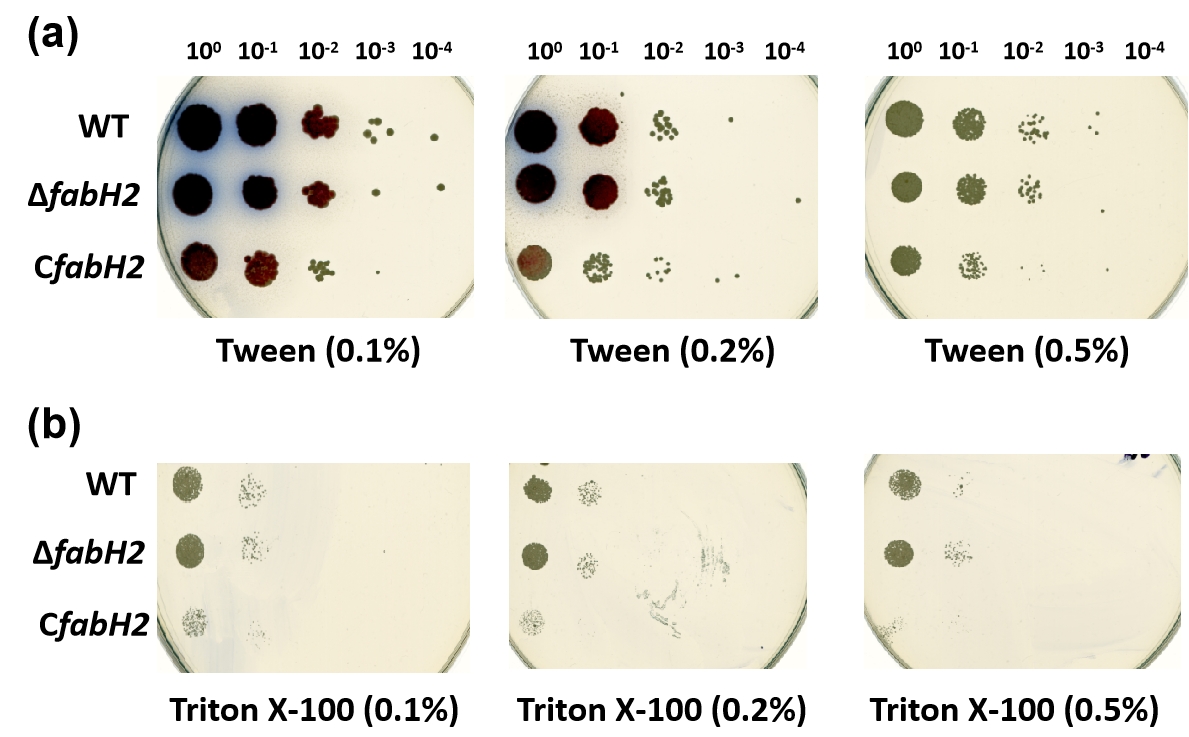


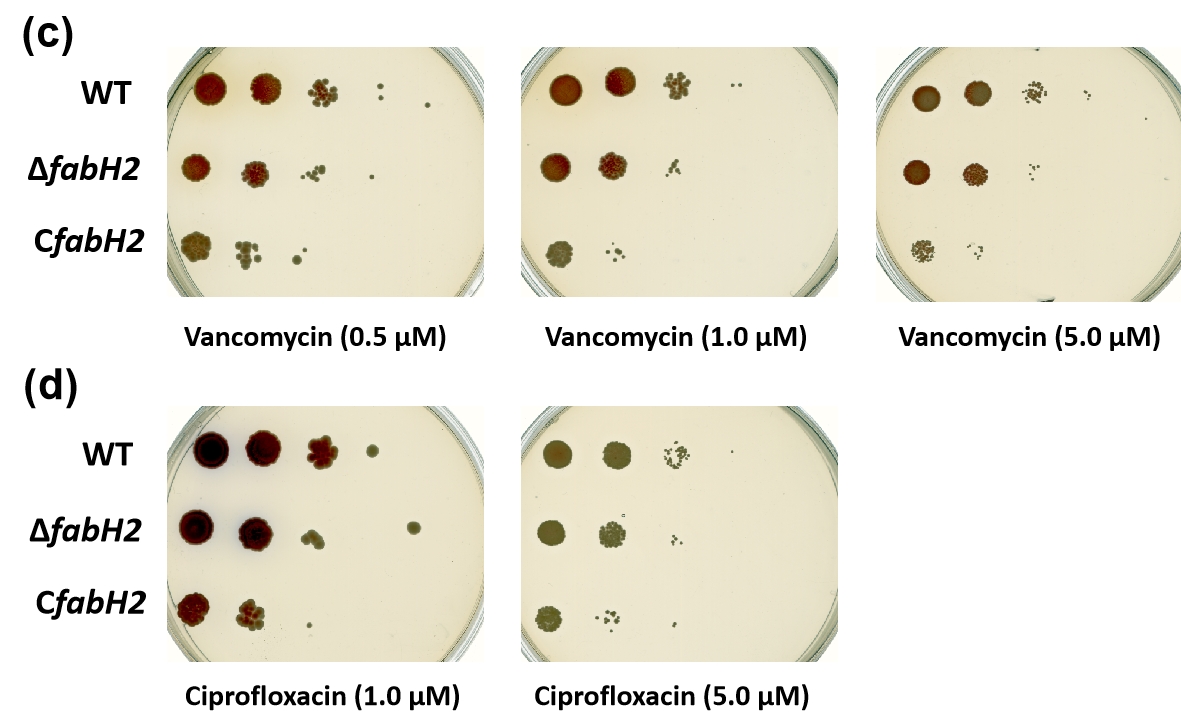


Figure S3 The complementary strain C*fabH2* showed higher sensitivity to detergents and antibiotics. The spores of WT , Δ*fabH2* and C*fabH2* were diluted to the same concentration (about 10^6^), which were further serially diluted and spotted on YEB plates supplemented with (**a**) Tween (0.1%-0.5%), (**b**) Triton X-100 (0.1%-0.5%), (**c**) Vancomycin (0.5-5 μM) and (d) Ciprofloxacin (1.0-5.0 μM), respectively. The experiments were performed in three biological replicates.


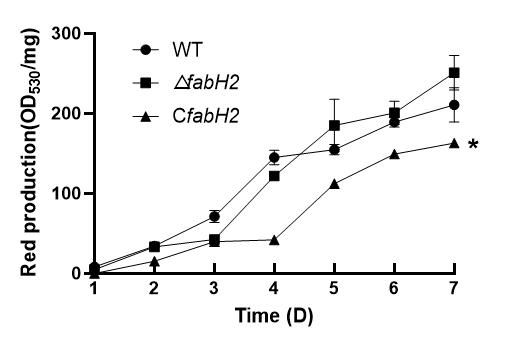


Figure S4 The RED production of different strains.


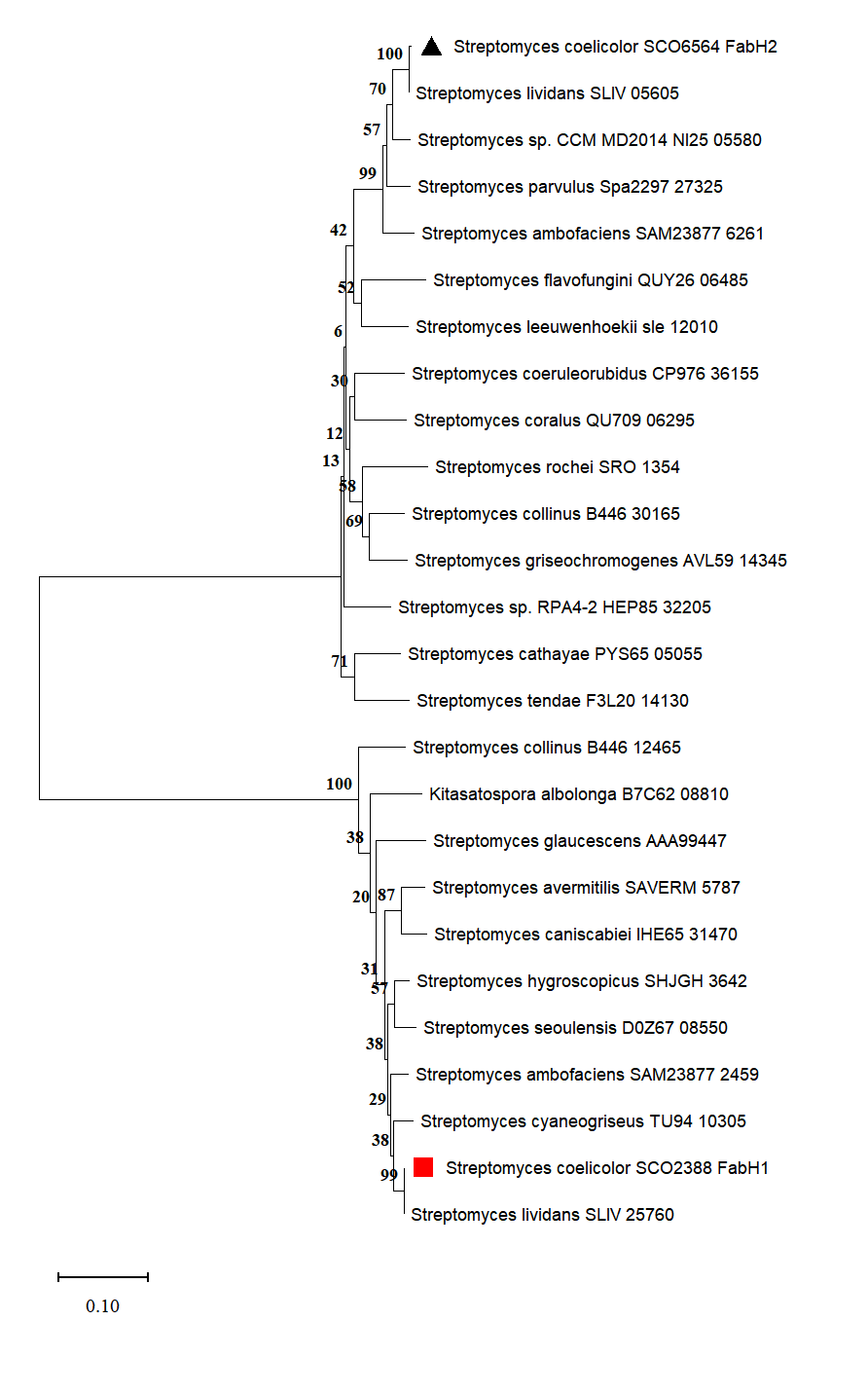


Figure S5 Phylogenetic analysis of *S. coelicolor* FabH1 and FabH2 homologs of different bacteria
