# Supplementary figures and images for: SCO6564, a novel 3-ketoacyl acyl carrier protein synthase III, contributes in fatty acid synthesis in Streptomyces coelicolor
Source: PLoS One. 2025 Feb 6;20(2):e0318258. doi: 10.1371/journal.pone.0318258 (PMC11801535; doi:10.1371/journal.pone.0318258)

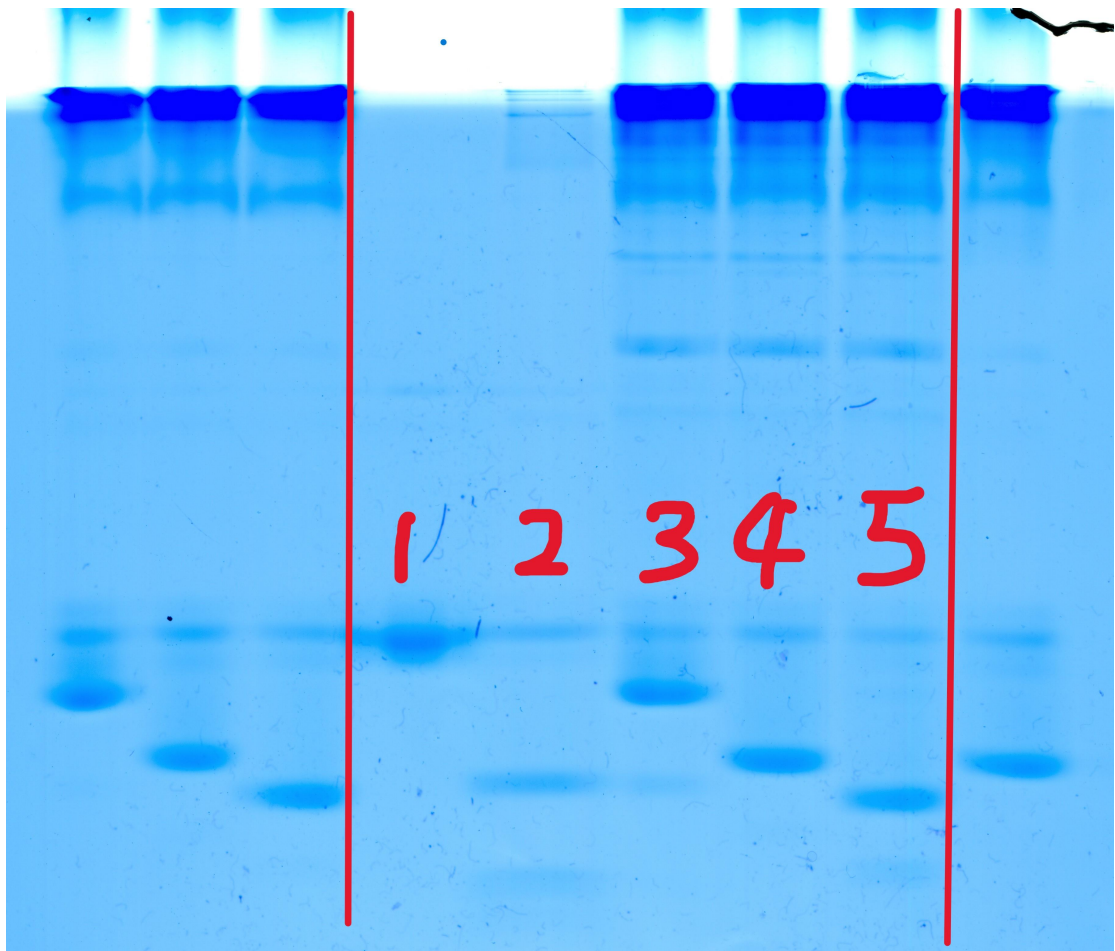

The original picture for Fig.3a

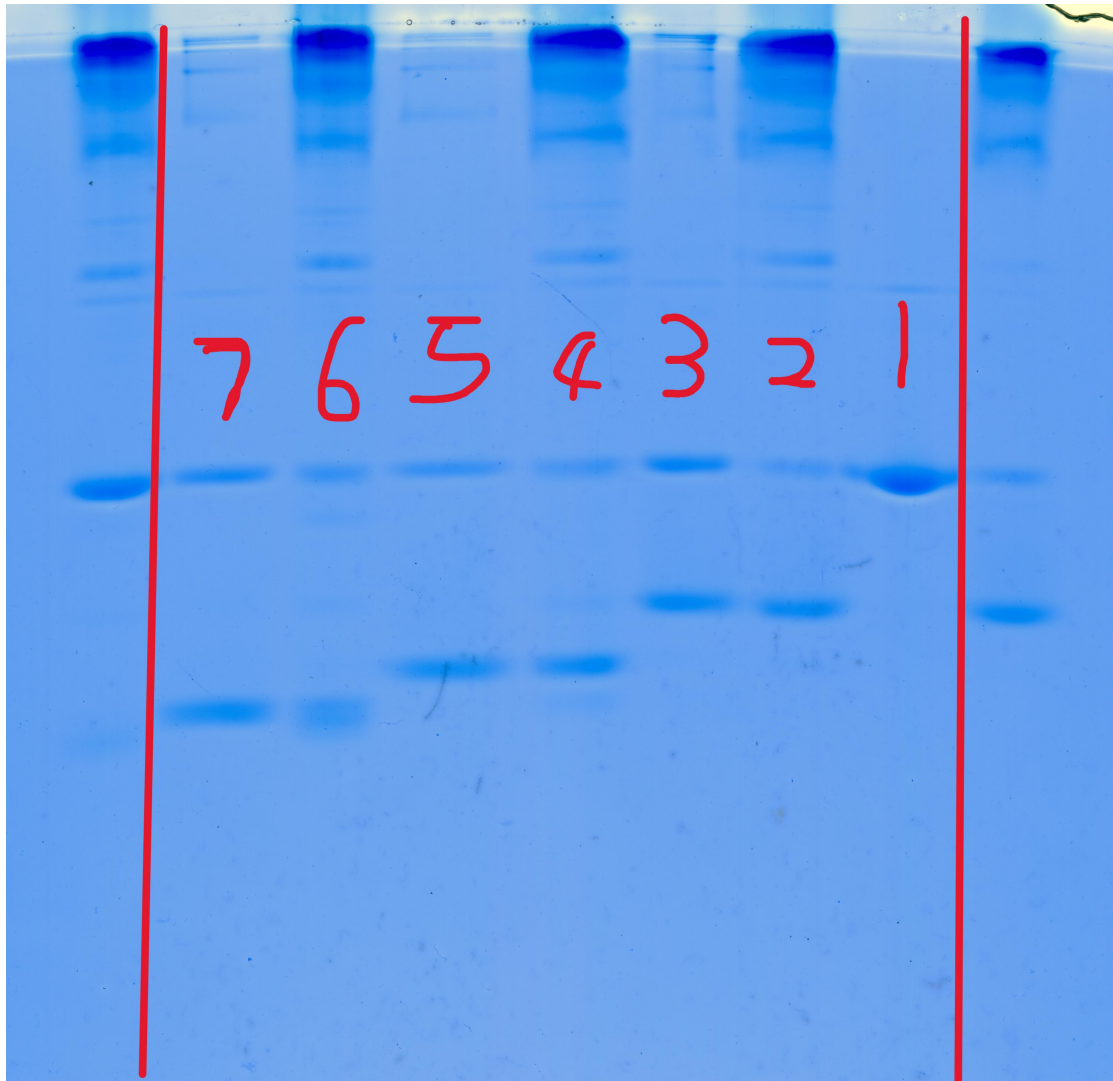

The original picture for Fig.3b

Supplement: S1 Raw images — (PDF) [file pone.0318258.s002.pdf]
